# Host immunogenetic variation and gut microbiome functionality in a wild vertebrate population

**DOI:** 10.1186/s40168-026-02341-9

**Published:** 2026-03-12

**Authors:** Chuen Zhang Lee, Sarah F. Worsley, Charli S. Davies, Jan Komdeur, Falk Hildebrand, Hannah L. Dugdale, David S. Richardson

**Affiliations:** 1 School of Biological Sciences, University of East Anglia, Norwich, Norfolk, UK; 2 Centre for Microbial Interactions, Norwich Research Park, Norwich, Norfolk, UK; 3 Groningen Institute for Evolutionary Life Sciences (GELIFES), University of Groningen, Groningen, The Netherlands; 4 Quadram Institute Bioscience, Norwich Research Park, Norfolk, UK; 5 Earlham Institute, Norwich Research Park, Norfolk, UK; 6 Nature Seychelles, Roche Caiman, Mahé, Republic of Seychelles

**Keywords:** *Acrocephalus sechellensis*, Metagenomics, Gut microbiome, Major histocompatibility complex, Wild population

## Abstract

**Background:**

The gut microbiome (GM) –important for host health and survival– is partially shaped by host immunogenetics. However, to date, no study has investigated the influence of host Major Histocompatibility Complex (MHC) genes on gut microbiome functionality in a wild population. Here we use a natural population of the Seychelles warbler (*Acrocephalus sechellensis*) to assess the effects of MHC genes on GM taxonomy and functionality using shotgun metagenomics.

**Results:**

Our results show that taxonomic GM composition was associated with MHC-II diversity and the presence of one specific MHC-I allele (*Ase-ua 7*). Specifically, MHC-II diversity was associated with decreased *Lactococcus lactis* and increased *Staphylococcus lloydii* abundance, while *Ase-ua 7* was linked to reduced *Enterococcus casselifavus* and *Gordonia sp* OPL2 but increased *Escherichia coli* and *Vulcaniibacterium thermophilum*. These taxonomic changes may reflect differences in MHC-mediated microbial recognition. In contrast, functional GM composition was significantly associated with increasing individual MHC-I diversity but not MHC-II diversity. In particular, increasing MHC-I diversity was associated with an increased prevalence of microbial defence genes but a reduced prevalence of microbial metabolism genes. Analysis also revealed that functional GM networks were more fragmented in high compared to low MHC-I diversity hosts.

**Conclusion:**

These results suggest that MHC variation (particularly at MHC-I) plays an important role in shaping both the taxonomy and function of the GM in wild vertebrates. In the Seychelles warbler, this results in trade-offs whereby there is an increase in microbial defence and a reduction in GM metabolic potential in individuals with higher MHC-I diversity. Thus, this work sheds light on the possible costs and benefits of maintaining a healthy microbiome, which is essential for understanding how the GM and immune system co-evolve.

Video Abstract

**Supplementary Information:**

The online version contains supplementary material available at 10.1186/s40168-026-02341-9.

## Introduction

The vertebrate gut microbiome (GM), a complex ecosystem of microorganisms inhabiting the gastrointestinal tract, is increasingly recognised as a critical determinant of host health and fitness [118]. However, the composition and function of the GM exhibit extensive variability across individuals, particularly in natural populations [26, 84, 109]. This variation has been attributed to a range of factors, such as diet, age, sex, location and host genetic variation [21, 111, 119].

A growing body of evidence links GM characteristics to host immunogenetic variation [23, 117]. Immune genes influence the immune system’s ability to recognise, tolerate, or eliminate microbial populations [20, 65]. Therefore, the immune system must maintain a balance – tolerating beneficial microbes while combating pathogens- to optimise host health [31, 97]. Furthermore, the GM also appears to play a role in the immune defences of the host, with GM dysbiosis (an imbalance in the composition of microbes) resulting in a reduction of host immune function, emphasising the interconnected nature of immune health and GM stability [54, 83].

The major histocompatibility complex (MHC) is a family of immune genes, forming part of the vertebrate acquired immune system [77]. These genes encode cell-surface glycoprotein receptor molecules that bind to antigens before presenting them to T lymphocytes and B cell receptors, which trigger an immune or tolerogenic response [5, 90]. The MHC has two main classes, MHC-I and MHC-II, based on the encoded receptors presenting intracellular or extracellular antigens, respectively [89, 90]. The role of the MHC in combating pathogens has been well-studied [49, 73], with the extraordinarily high polymorphism of MHC genes observed in natural populations thought to be driven by pathogen-mediated selection mechanisms and sexual selection [94]. Individual MHC variation determines the range of microbial antigens recognised by the immune system [6], and is associated with variation in commensal gut microbial communities [21, 53, 92, 99]. Thus, different MHC genotypes could shape individual GM variation by initiating immune responses to potentially pathogenic microbes while maintaining beneficial species [91, 92].

Previous studies examining the impact of MHC variation on the GM in wild animals using 16S metabacoding, have reported mixed findings. Several have found that increased MHC diversity is associated with decreased microbiome diversity [6, 58, 101] but others associated it with increased [41] or unchanged GM diversity [21, 68]. Similarly, some studies have observed shifts in taxonomic composition with MHC diversity [6, 41, 68], while others have not [21, 29, 30, 101]. Additionally, the presence/absence of specific MHC alleles (rather than the overall diversity of alleles) has been found to be correlated with GM taxonomic composition [6, 21].

The functional composition of the GM – represented through microbial genes – could provide a more direct representation of host-microbe interactions [112]. However, the consequences of MHC variation for GM functionality have remained underexplored so far [31]. Many microbes share genes and, consequently, have similar functional roles [64]. Therefore, changes in microbial taxa do not always result in altered GM function – i.e. there is functional redundancy [64, 112]. Functional redundancy refers to the ecological concept that multiple species within an ecosystem can perform similar roles, encoding the same gene and/or different genes with the same function, buffering against species loss [64, 112]. Studying functionality is important for understanding if and how host genetic variation interacts with the GM to influence host fitness and evolutionary trajectories [112]. Most studies on MHC and microbial functionality rely on 16S metabarcoding markers and infer function based on known microbial taxa-function association [32, 33, 96, 103]. However, in less studied systems, such as wild animals, functional inferences from 16S metabarcoding markers may lead to misassignments due to the lack of representation of the specific microbes observed in existing databases [96, 100].

In humans and transgenic captive mice (*Mus musculus*), MHC haplotype is associated with GM functional composition [3, 8]. However, captive/domesticated populations often harbour greatly reduced genetic variation and microbial diversity compared to natural populations [106],thus, these results may not be transferable to wild systems. To our knowledge, the only pioneering study of host MHC and GM function in a wild animal so far used 16S functional inferences [68], which are likely to lead to inaccuracies [96, 100]. Shotgun metagenomics or transcriptomic approaches are needed to accurately determine gut microbiome function in response to host MHC variation in wild animal populations.

Here, we investigate the relationship between MHC and GM variation in a population of Seychelles warblers (*Acrocephalus sechellensis*). Despite reduced neutral genetic variation due to past population bottlenecks [95], the Seychelles warbler has maintained variation (albeit reduced) at MHC-I and MHC-II loci [21, 39, 88]. Furthermore, one specific MHC allele (*Ase-ua 4*) and MHC-I diversity overall have been positively correlated with survival and reproductive success [12, 87]. A previous 16S-based analysis of this population has demonstrated that MHC alleles are associated with changes in bacterial GM taxonomic diversity and composition [21]. An analysis of the fungal mycobiome also reported changes in species diversity and composition associated with MHC alleles and MHC-I diversity, respectively [110]. Since these studies, we have greatly expanded our sample size, identified key environmental control variables, and conducted shotgun sequencing for metagenomic analysis [59].

We leverage a powerful combination of both 16S rRNA metabarcoding (larger sample size) and shotgun metagenomics to provide a comprehensive and high-resolution assessment of the association between host MHC variation and both taxonomic and, importantly, functional components of the bacterial GM in adult Seychelles warblers. First, we test if GM taxonomic diversity and composition correlate with MHC-I and MHC-II diversity or alleles. Next, we test if GM functional diversity and functional composition are associated with this MHC variation. Finally, we assess the role of functional redundancy in preserving the functionality of the GM despite changes in host MHC variation.

## Methods

### Study system

The population of insectivorous Seychelles warblers on Cousin Island (29 ha; 04° 20′ S, 55° 40′ E) has been extensively monitored since 1985 [13, 51] Two field seasons are undertaken annually from ca. January to March (minor) and June to September (major). Each season, as many individuals as possible are caught in the nest (chicks) or using mist nets and sampled (see below). New individuals are marked with a British Trust for Ornithology (BTO) metal ring and a unique combination of three colour rings, allowing them to be monitored throughout their lives. Almost every bird (> 96%) on Cousin has been marked this way since 1997 [81, 85]. Age is calculated based on fledge or hatch dates, or eye colour at first catch [50]. This population includes ca. 320 individuals in approximately 115 territories [38, 52].

### Sample collection

Faecal sample collection, storage, DNA extraction, library preparation and sequencing were conducted between 2017 and 2023, as part of (and described in full in) previous studies using 16S rRNA metabarcoding [21, 108, 112] and shotgun metagenomics [59]. In brief, caught birds were placed in a flat-bottom paper bag with a sterilised weigh boat under a metal grate, allowing faeces to drop to the weigh boat, while minimising contact with the birds’ surface. Faecal matter was transferred into a sterile microcentrifuge tube containing 1 mL of absolute ethanol, stored at 4 °C during fieldwork and then at −80°C for long-term storage at the University of East Anglia (UEA). The time-of-day (minutes after sunrise; 06:00 AM) of sampling was recorded. Each season, control samples were also taken from the hands of fieldworkers using a sterile swab and stored in the same manner. A small (ca 25 μL) blood sample was also collected from each bird via brachial venepuncture and stored in 0.7 ml of absolute ethanol at 4 °C. Samples may be collected from the same individual in different field seasons; thus, the identity of each individual sampled (Bird ID) is recorded and used to control for these repeated measures in statistical analyses. In total, 149 unique bird IDs were sampled, of which 72 had repeated samples.

### Molecular genotyping

Total genomic DNA was extracted from blood samples using the DNeasy Blood and Tissue kit (Qiagen, Crawley, UK) according to the manufacturer’s protocol. All Individuals were genotyped using up to 30 polymorphic microsatellite loci and 3 sexing markers (following [37, 86, 93]) as part of the ongoing determination of parentage and pedigree within this population [82]. Individual genome-wide heterozygosity (Hs) at these neutral loci was calculated using *genhet* 3.1 in R 4.33 [19, 80] as per [113].

Sequencing of amplified MHC-I exon 3 and MHC-II exon 2 variants using Illumina MiSeq technology had already been undertaken for 314 warblers [21]. All confirmed variants (20 MHC-I and 14 MHC-II,hereafter termed alleles) at each of these MHC regions (which contain 4 replicated loci) [46, 88] were used to calculate individual MHC diversity. However, due to statistical power limitations, only alleles present in > 5% and < 95% of individuals were included in presence/absence analyses. Two MHC-I alleles (*Ase-ua1* and *Ase-ua10* were co-occurring; thus, only one of them, Ase-ua1, was retained for downstream analyses. Therefore, nine MHC-I alleles and three MHC-II alleles were used in the presence/absence statistical analyses. Each allele in the presence/absence analysis translates to a unique amino acid sequence with different antigen-binding properties [21]. Pairwise p-distance of MHC alleles was also calculated based on the percentage of different amino acids. The average pairwise p-distance was then calculated per individual to represent functional diversity of the MHC (i.e. MHC divergence).

### Gut microbiome screening

Microbial DNA from faecal samples was extracted using the DNeasy PowerSoil Kit (Qiagen, Crawley, UK) and a modified version of the manufacturer’s protocol (described in detail [21]). Samples were randomised across extractions to minimise batch effects.

Faecal DNA samples were submitted for 16S rRNA amplicon sequencing. Amplicon sequencing libraries were generated using the V4 primers 515 F (5'TGCCAGCMGCCGCGGTAA3’) and 806R (5’GGACTACHVGGGTWTCTAAT3’). Libraries were sequenced across seven batches using 2× 250bp, paired-end sequencing on an Illumina MiSeq Platform (see [21, 108, 112]). Control samples were also extracted, library prepped and sequenced the same way (*n* = 21 hand controls, 15 negative controls, and 10 positive ZymoBIOMICS Microbial Community Standard (D6300) controls).

Faecal DNA samples underwent library preparation using the LITE protocol [75] and were sequenced using 2 × 150 bp, paired-end shotgun metagenomic sequencing in two runs on an Illumina NovaSeq X platform (see [59]). Hand controls (*n* = 6) and positive controls (*n* = 3, two ZymoBIOMICS Microbial Community Standard (D6300), and one ZymoBIOMICS Fecal Reference with TruMatrix™ Technology (D6323)) were also prepped and sequenced as part of the metagenomic samples sequencing.

### Bioinformatics

Read processing for 16S metabarcoding was performed as previously described [108, 112]. Briefly, 16S rRNA reads were processed using QIIME2 2019.10,reads were truncated, filtered, and classified into amplicon sequencing variants (ASV) using DADA2 [15]. ASVs were then taxonomically assigned using the naïve-Bayes classifier on the SILVA 132 reference database for 16S rRNA gene sequences [7]. These ASVs were then imported into R 4.3.3 using *phyloseq* 1.46.0 [15, 66], then filtered to remove non-bacterial sequences, reads unassigned to phylum level, and potential contaminants (based on hand controls). Rarefaction curves were constructed with *iNEXT* version 3.0.1 with the default 50 bootstrap replications [18], reaching an asymptote at 8000 reads, indicating sample completeness (Figure S1A). In addition, 27 faecal samples with < 8000 reads were removed and ASVs with < 50 reads across all samples were also removed.

Shotgun metagenomic sequence processing was performed using *MATAFILER* [43] as previously described [59]. Host reads were removed by mapping reads with *Kraken2* 2.1.3 to the Seychelles warbler genome (unpublished; complete BUSCO = 96.0% with a total length = 1,081,018,985 bp), followed by read quality filtering using *sdm* 2.14 beta; minimum sequence length of 50, minimum average quality of 27 [44, 107], an average of 21% (± 0.07SE) reads were removed. After trimming, two samples and five hand controls were removed because they did not have enough reads for metagenome assembly. An average of 20,481,040 (SD = 13,718,305) paired-end reads per sample were retained for de novo metagenome assembly using *MEGAHIT* 1.2.9 with default parameters and kmer-list of 25,43,67,87,111,131 [61]. Using the resulting assemblies, genes were predicted using Prodigal 2.6.3 [47] and clustered into gene catalogues (95% identity). Genes were functionally annotated using *eggNOGmapper* 2.1.12 with default parameters and the eggNOG database 4 [16, 79]. Functional categories were also assigned to each functional annotation based on the cluster of gene orthologs (COG) database [98]. Metaphlan4 assignments were used to taxonomically assign shotgun sequencing reads. Rarefaction curves were constructed for metagenomics taxonomy and functional reads with *iNEXT* version 3.0.1 with the default 50 bootstrap replications [18] and showed an asymptote and sample completeness at 5,500 and 100,000 reads, respectively (Figure S1B-C).

### Statistical analysis

Adult warblers with both microbiome and MHC data were analysed. For 16S analysis, 253 samples from 149 individuals were included in this study. Of these, 99 samples from 57 adult individuals also had GM shotgun metagenomic data. Individuals carried a mean of 5.13 (SE: 0.088, range 2–7) MHC-I alleles and 2.88 (SE: 0.060, range 1–5) MHC-II alleles. Due to the low number of samples for which we had shotgun metagenomic data, we had to limit the number of predictor variables (i.e. < 9) in each model to avoid overfitting and unreliable estimates. Thus, for MHC diversity and MHC divergence models, all control variables (see below) were included, but we first used the 16S metabarcoding dataset to shortlist which genetic metrics (including specific MHC alleles) should be included in the shotgun metagenomic models. Unless stated otherwise, all statistical analyses were conducted in R 4.3.3 in R Studio 2024.12.0 + 467 [78, 80] and linear mixed effect (LMMs) and generalised linear mixed effect models (GLMMs) were constructed using *lme4* 1.1–35.5 [1].

### GM diversity

#### 16S rRNA metabarcoding diversity

Reads were rarefied to 8,000 reads with the *rarefy_even_depth* function in *vegan* 2.6.6 (Oksanen [71]) – the point at which the number of ASVs identified reached an asymptote in rarefaction curves (Figure S1A)—before calculation of alpha diversity metrics. Both ASV richness and Shannon diversity were calculated for each sample using *phyloseq* 1.46.0 [66].

A GLMM with a negative binomial distribution was constructed with ASV richness as a response variable. An LMM with a Gaussian distribution was used to model Shannon diversity. Hereafter, all 16S models were tested with the same set of variables (described below) with either MHC alleles or MHC diversity as the response unless stated otherwise. MHC-I and MHC-II diversity (i.e. the number of alleles per individual) were included as predictors, along with genome-wide heterozygosity, age, season, sample year, sex, sample days at 4 °C, and time of day sampled, as fixed-term control variables and bird ID as a random effect. Quadratic effects of MHC-I diversity and MHC-II diversity were included to test if an intermediate number of alleles influenced GM characteristics, but were dropped sequentially if not significant (least significant first) to allow interpretation of the main terms. Standardised effect sizes of each fixed effect were determined using partial R^2^ with *r2beta*() function in r2glmm version 0.1.3 [48].

To determine if specific MHC alleles were shaping the GM, a second model was constructed using the presence/absence of MHC-I (*Ase-ua 1, Ase-ua 3, Ase-ua 4, Ase-ua 5, Ase-ua 6, Ase-ua 7, Ase-ua 8, Ase-ua 9, Ase-ua 11*) and MHC-II alleles (*Ase-dab 3*, *Ase-dab 4*, *Ase-dab 5*) as predictors in place of MHC diversity.

#### Metagenomic taxonomic diversity

Metaphlan4 assignments were rarefied to 5,500 reads (Figure S1B) – prior to alpha diversity analysis. A GLMM with a negative binomial distribution was then used to model species richness, and an LMM was used to model Shannon diversity. All metagenomics analyses were performed with the same structure (described below, i.e. MHC diversity models included all terms, while MHC presence/absence models only included genetic variables that were identified as significant in the corresponding 16S analysis).

A second model with the presence/absence of specific MHC alleles (identified as significant in the 16S metabarcoding model above) was constructed.

#### Metagenomic functional diversity

Functional gene annotations (determined using eggNOG mapper described above) were rarefied to 100,000 reads (Figure S1C) before functional alpha diversity analysis. Scaled exponentially transformed functional gene richness and exponentially transformed functional Shannon diversity were modelled separately with LMMs, with either the MHC diversity or the presence/absence of MHC alleles, along with genome-wide heterozygosity and environmental control variables (as described for 16S analyses above). An additional model including the presence/absence of all MHC alleles was constructed, as functional GM characteristics may respond differently to MHC variation than GM taxonomy. However, because the metagenomic dataset (*N* = 99 from 57 individuals) contains fewer samples than the 16S metabarcoding (*N* = 253 from 149 individuals), incorporating all alleles risks over-parameterising the model. Therefore, a reduced model was also constructed that only included the specific MHC alleles identified as significant in the 16S metabarcoding analysis to evaluate the robustness of the results.

### GM composition

#### 16S rRNA metabarcoding composition

Unrarefied reads were used. Rare ASVs (< 5% prevalence) were removed prior to analysis, and a centred log ratio (CLR) transformation was applied to the remaining ASV abundances using *microbiome* 1.24.0 [60]. Pairwise Aitchison distances (i.e. composition differences) among GM samples were then modelled via a PERMANOVA using the *adonis2*() function with by = “margin” in *vegan* 2.6.6 with 9999 permutations. A blocking effect of bird ID was included to account for repeated sampling (Oksanen [71]). The first PERMANOVA model included MHC diversity, genome-wide heterozygosity, age, season, sample year, sex, days at 4 °C and time of day as predictors. The second PERMANOVA model had the presence/absence of individual MHC alleles instead of MHC diversity. Heterogeneity of group dispersions among significant MHC alleles, seasons, and sampling years was tested using *betadisper*() and *permutest*() functions in vegan. Both these and all subsequent GM composition models were set up in the same way, tested for heterogeneity of dispersion, and visualised with a PCA generated in *phyloseq* 1.46.0 [66] unless stated otherwise.

#### Metagenomic taxonomic composition

Rare species were removed (< 5% prevalence), the remaining unrarefied reads were CLR transformed and used in a PERMANOVA to identify differences in taxonomic composition associated with MHC variation (as described for 16S analysis above). For the MHC alleles model, only genetic predictors identified as significant in the 16S rRNA metabarcoding composition analysis were included, along with all control variables (as described for 16S analyses above).

#### Metagenomic functional composition

Rare functional genes (< 5% prevalence) were removed, and the remaining unrarefied reads were CLR transformed and used in a PERMANOVA to test for differences in functional composition linked to MHC variation (as described for metagenomic taxonomic composition analyses above). For the MHC alleles model, all alleles were included, but that could overparameterize the models; thus, an additional model was created with only genetic predictors identified as significant in the 16S rRNA metabarcoding composition analysis.

### Differential abundance analyses

#### Differential abundance of metagenomic taxonomic species

Differential abundance tests were carried out using *ALDEx2* 1.34.0 [28]. Only common species (> 10% prevalence and > 0.001% abundance resulting in 49 metagenomic identified species) were included. Abundances were CLR transformed as part of the *ALDEx2* method [28]. Genome-wide heterozygosity, MHC-I and MHC-II diversity, as well as significant variables identified in the metagenomic taxonomic composition analysis were included as predictors.

#### Differential abundance of metagenomic functional genes

Abundances of common functional genes (> 50% prevalence, > 0.1% abundance resulting in 94 eggNOG members) were CLR-transformed using *ALDEx2* 1.34.0 [28] and included in this analysis. Predictors were included as above but based on significant variables in metagenomic functional composition analysis. KEGG orthologs and pathways were assigned manually to significant functional genes by referring to the KEGG website (https://www.kegg.jp/kegg/kegg2.html). The relative abundance (determined using CLR transformation) of each significant functional category was tested with a GLMM to directly test for trade-offs between categories. Genome-wide heterozygosity, MHC-I diversity, MHC-II diversity, as well as significant variables identified in the metagenomic functional composition analysis were included as predictors. Bird ID was included as a random effect.

### Network analysis

#### Network analysis of metagenomic taxonomic species

Networks of metagenomic taxonomic species were constructed with SParse InversE Covariance Estimation for Ecological Association Inference (SPIEC-EASI) version 1.0.7 [55]. The samples were split into two categories based on being higher or lower than the average MHC diversity in the population (see above): low (< 6) and high (≥ 6) MHC-I diversity, or low (< 3) and high (≥ 3) MHC-II diversity. The raw counts of common bacterial species (> 10% prevalence and > 0.1% abundance) were used as inputs, with SPIEC-EASI applying a CLR transformation. Common species were used to capture relevant, stable GM species and minimise the influence of rare taxa [25]. The number of nodes (species), the number of edges (interaction between species), the average number of connections per node, modularity and negative-to-positive ratios were calculated. The difference in edge density, modularity, and positive edge percentage between low and high MHC diversity were tested with the *netCompare*() function in NetCoMi 1.2.0 [76]. The networks were then plotted with the *ggnet2* function in ggnet 0.1.0 [11]. Nodes were coloured by Phylum, and size was based on mean abundance per species.

#### Network analysis of metagenomic functional genes

Networks were constructed as described above but using common metagenomic functional genes (eggNOG genes) (> 50% prevalence, > 0.1% abundance) instead of metagenomic species.

### MHC divergence vs. MHC diversity

In response to reviewer’s comments, we also tested whether using an alternate measure of MHC diversity, i.e. MHC divergence [22, 40], affected our interpretation of how the GM was associated with MHC variation. To do this, we added complementary models that replace MHC diversity with MHC divergence. This MHC comparison is useful because it captures different biological aspects of the MHC: MHC diversity reflects the number of MHC alleles that are present, whereas MHC divergence quantifies how functionally different those alleles are from one another [22]. In each case, model performance was compared using Akaike Information Criterion (AIC) values calculated with the *AIC*() function. An AIC difference > 7 between two models is considered evidence for improved model fit [14]. Given that the results were qualitatively very similar between models using the different metrics (see results), we have added the detailed methods and results for this additional analysis in the supplementary and report the results of MHC diversity models in the main text.

## Results

### GM diversity

#### 16S rRNA metabarcoding diversity

GM alpha diversity (Shannon diversity or richness) was not significantly associated with MHC-I or MHC-II diversity (Table 1A, Table S1A & S2A & S3A). The presence of the MHC-I allele *Ase-ua 11*—but no other MHC allele—was significantly positively associated with 16S richness (Table 1B, Table S1B & S2B & S3B). No alleles were associated with Shannon diversity.

**Table 1:** The relationship between gut microbiome alpha diversity (richness) and variation in host (A) Major histocompatibility complex (MHC) diversity and (B) the presence/absence of specific MHC alleles in adult Seychelles warblers. Generalised linear mixed models with a negative binomial distribution were used for 16S ASV diversity (*N* = 253 samples from 149 individuals) and metagenomics taxonomy diversity (*N* = 99 samples, 57 individuals), and linear mixed models were used for metagenomics functional diversity (*N* = 99 samples, 57 individuals). Reference categories for categorical variables were as follows: Female (sex), winter (season), 2017 (Sample year), and absent (in all MHC alleles). Significant (*P* < 0.05) variables are shown in bold. Shannon diversity results are similar and shown in Supplementary Table S1

Predictors	16S ASV diversity				Metagenomics taxonomic diversity				Metagenomics functional diversity			
	Est	SE	z	P	Est	SE	z	P	Est	SE	t	P
A) MHC Diversity												
(Intercept)	**5.37**	**0.32**	**16.82**	**< 0.001**	**3.23**	**0.70**	**4.62**	**< 0.001**	**1.06**	**0.45**	**2.39**	**0.02**
Heterozygosity	−0.19	0.21	−0.93	0.35	0.43	0.43	1.02	0.31	−0.13	0.28	−0.46	0.65
MHC-I Diversity	0.04	0.03	1.26	0.21	−0.01	0.06	−0.18	0.85	0.07	0.04	1.60	0.12
MHC-II Diversity	−0.01	0.04	−0.23	0.82	−0.14	0.08	−1.83	0.07	−0.02	0.06	−0.26	0.79
Age	−0.02	0.02	−1.28	0.20	−0.04	0.03	−1.12	0.26	**−0.04**	**0.02**	**−2.07**	**0.04**
Season (summer)	0.04	0.12	0.30	0.76	0.19	0.22	0.90	0.37	−0.04	0.14	−0.26	0.79
Sex (male)	**−0.22**	**0.09**	**−2.51**	**0.01**	0.15	0.17	0.90	0.37	−0.04	0.12	−0.31	0.76
Days at 4°C	−0.01	0.09	−0.05	0.96	0.02	0.19	0.13	0.90	−0.07	0.10	−0.63	0.53
Time of day	0.02	0.08	0.19	0.85	0.26	0.18	1.46	0.15	−0.06	0.10	−0.60	0.55
Sample Year (2018)	−0.03	0.13	−0.27	0.79	0.01	0.27	0.05	0.96	0.13	0.16	0.84	0.40
Sample Year (2019)	0.14	0.16	0.87	0.39	−0.29	0.37	−0.79	0.43	−0.03	0.21	−0.13	0.90
Sample Year (2020)	**0.48**	**0.21**	**2.29**	**0.02**	−0.02	0.45	−0.05	0.96	0.08	0.26	0.31	0.76
Sample Year (2021)	0.21	0.16	1.32	0.19	0.00	0.37	−0.01	0.99	0.07	0.20	0.33	0.75
Sample Year (2022)	0.14	0.16	0.88	0.38	0.45	0.31	1.44	0.15	0.31	0.18	1.70	0.09
Sample Year (2023)					0.08	0.36	0.22	0.83	−0.05	0.21	−0.25	0.81
B) Presence/absence of MHC alleles												
(Intercept)	**5.66**	**0.33**	**17.37**	**< 0.001**	**3.24**	**0.29**	**11.15**	**< 0.001**	**1.48**	**0.49**	**3.02**	**0.004**
Heterozygosity	−0.09	0.21	−0.42	0.68					−0.09	0.32	−0.29	0.773
Ase-dab3	0.29	0.15	1.95	0.05					0.37	0.23	1.62	0.116
Ase-dab4	−0.27	0.15	−1.76	0.08					−0.22	0.24	−0.89	0.378
Ase-dab5	0.18	0.16	1.15	0.25					0.00	0.27	−0.01	0.995
Ase-ua1	0.12	0.18	0.64	0.52					−0.05	0.26	−0.20	0.844
Ase-ua3	−0.11	0.19	−0.60	0.55					−0.02	0.29	−0.08	0.935
Ase-ua4	−0.19	0.14	−1.37	0.17					−0.16	0.25	−0.65	0.520
Ase-ua5	−0.09	0.18	−0.52	0.60					0.02	0.29	0.08	0.938
Ase-ua6	−0.18	0.17	−1.04	0.30					−0.16	0.27	−0.60	0.555
Ase-ua7	−0.16	0.20	−0.80	0.43					−0.15	0.27	−0.54	0.593
Ase-ua8	−0.04	0.14	−0.29	0.78					0.16	0.23	0.70	0.491
Ase-ua9	−0.07	0.17	−0.39	0.70					−0.29	0.29	−1.00	0.324
Ase-ua11	**0.38**	**0.18**	**2.07**	**0.04**	0.00	0.16	0.01	0.99	0.45	0.28	1.59	0.121
Age	−0.03	0.02	−1.63	0.10	−0.06	0.03	−1.67	0.10	−0.05	0.03	−1.70	0.096
Season (summer)	0.04	0.12	0.31	0.75	0.09	0.22	0.42	0.68	0.04	0.14	0.25	0.803
Sex (male)	**−0.25**	**0.09**	**−2.88**	**< 0.001**	0.12	0.17	0.75	0.45	−0.09	0.13	−0.66	0.512
Days at 4°C	−0.03	0.09	−0.33	0.75	−0.03	0.19	−0.16	0.88	−0.08	0.11	−0.72	0.476
Time of day	0.08	0.08	0.92	0.36	0.27	0.18	1.48	0.14	−0.06	0.11	−0.53	0.600
Sample Year (2018)	−0.06	0.12	−0.45	0.65	0.08	0.28	0.30	0.76	0.14	0.17	0.81	0.420
Sample Year (2019)	0.09	0.16	0.56	0.58	−0.13	0.37	−0.35	0.73	−0.13	0.22	−0.60	0.551
Sample Year (2020)	**0.42**	**0.21**	**2.02**	**0.04**	−0.08	0.46	−0.18	0.86	0.09	0.27	0.35	0.729
Sample Year (2021)	0.21	0.16	1.30	0.19	0.14	0.37	0.38	0.71	0.09	0.21	0.42	0.678
Sample Year (2022)	0.12	0.15	0.79	0.43	0.57	0.32	1.79	0.07	0.28	0.20	1.35	0.181
Sample Year (2023)					0.29	0.37	0.78	0.43	−0.15	0.24	−0.64	0.526

#### Metagenomic taxonomic diversity

Taxonomic alpha diversity (Shannon diversity or richness) calculated using shotgun metagenomics data was not associated with MHC-I or MHC-II diversity (Table 1A, Table S1A & S2A & S3A), nor with the presence/absence of *Ase-ua 11* (the MHC variant identified in the 16S analysis above (Table 1B, Table S1B & S2B & S3B)).

#### Metagenomic functional diversity

Functional alpha diversity (Shannon diversity or richness) of gene annotations derived from shotgun metagenomics data was not associated with MHC-I or MHC-II diversity (Table 1A, Table S1A & S2A & S3A), nor with MHC alleles (Table 1B, Table S1B & S2B & S3B, Table S4).

### GM composition

#### 16S rRNA metabarcoding composition

**Fig. 1:**
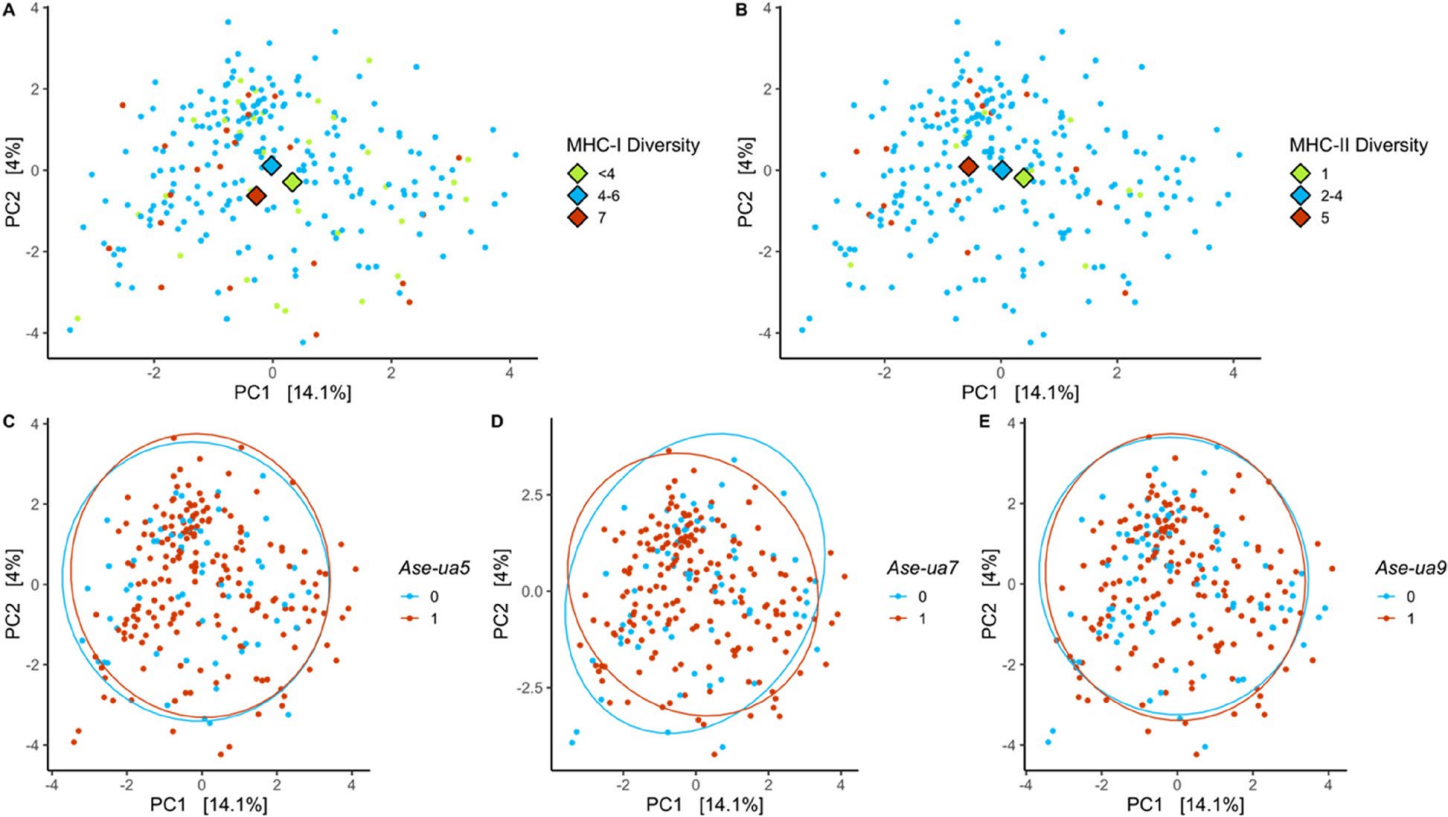
Principal Component Analyses (PCA) of gut microbiome compositional variation determined using 16S rRNA metabarcoding of adult Seychelles warbler faecal samples in relation to MHC-I diversity (**A**), MHC-II diversity (**B**), and the presence/absence (1/0) of MHC-I allele *Ase-ua 5 *(**C**), MHC-I allele *Ase-ua 7 *(**D**), MHC-I allele *Ase-ua 9 *(**E**). *N* = 253 from 149 birds. Large diamonds represent the group centroids. For clarity, samples were grouped into discrete categories for plotting. In plots **A**-**B**, the coloured points represent low (green), medium (blue), and high (red) MHC diversity. In plots **C**-**E**, blue = absence, red = presence of the allele. Ellipses of 95% confidence intervals of each group are drawn around the points. Principal components 1 and 2 explained 14.1% and 4% of the variation in gut microbiome structure, respectively

16S GM composition was associated with both a quadratic function of MHC-I diversity and of MHC-II diversity (Table 2A, Fig. 1A-B). It was also associated with season, sample year, days at 4 °C, and time of day (Table 2A) but not with genome-wide heterozygosity, age, and sex.

**Table 2:** PERMANOVA analyses of gut microbiome composition in relation to individual major histocompatibility complex (MHC) characteristics in adult Seychelles warblers. Performed using Euclidean distance matrices of CLR-transformed abundances of (I) 16S amplicon sequencing variants (ASV) composition, (II) metagenomic taxonomic composition, (III) metagenomic functional gene composition categories. Separate models included (A) MHC diversity and (B) the presence/absence of MHC alleles. Significant predictors (*p* < 0.05) are in bold. *N* = 253 samples from 149 individuals were included in the 16S metabarcoding analyses. *N* = 99 samples from 57 individuals were used for analyses of metagenomic taxonomic and functional composition. Bird ID was included as a blocking factor

Predictor	(I) 16S ASV composition				(II) Metagenomics taxonomic composition				(III) Metagenomics functional gene composition			
	*df*	R^2^	F	*p*	*df*	R^2^	F	*p*	*df*	R^2^	F	*p*
A) MHC Diversity												
Heterozygosity	**1**	0.003	0.719	0.067	**1**	**0.008**	**0.797**	**0.030**	1	0.015	1.485	0.052
MHC-I Diversity	**1**	**0.003**	**0.919**	**0.007**	1	0.010	1.034	0.626	**1**	**0.014**	**1.423**	**0.045**
MHC-I Diversity^2	**1**	**0.004**	**0.951**	**0.006**	-				-			
MHC-II Diversity	**1**	**0.003**	**0.839**	**0.036**	**1**	**0.009**	**0.925**	**0.034**	1	0.011	1.069	0.642
MHC-II Diversity^2	**1**	**0.003**	**0.913**	**0.028**	-				-			
Age	1	0.003	0.918	0.930	1	0.015	1.625	0.528		0.010	0.976	0.893
Season	**1**	**0.007**	**1.955**	**< 0.001**	**1**	**0.019**	**1.995**	**0.001**	1	0.014	1.368	0.155
Sample Year	**5**	**0.038**	**2.023**	**< 0.001**	**6**	**0.085**	**1.492**	** < 0.001**	6	0.063	1.044	0.202
Sex	1	0.003	0.914	0.870	**1**	**0.012**	**1.228**	** < 0.001**	1	0.012	1.206	0.185
Days at 4°C	**1**	**0.010**	**2.618**	**0.008**	1	0.011	1.116	0.494	**1**	**0.014**	**1.394**	**0.015**
Time of day	**1**	**0.010**	**2.525**	**0.001**	**1**	**0.017**	**1.773**	** < 0.001**	1	0.013	1.325	0.168
B) Presence/absence of MHC alleles												
Heterozygosity	1	0.003	0.679	0.246	**-**				-			
* Ase-dab3*	1	0.004	1.004	0.999	-				1	0.009	0.853	0.406
* Ase-dab4*	1	0.003	0.894	0.371	-				1	0.013	1.336	0.787
* Ase-dab5*	1	0.004	0.991	0.740	-				1	0.007	0.744	0.634
* Ase-ua1*	1	0.004	1.107	0.084	-				1	0.012	1.151	0.125
* Ase-ua3*	1	0.004	0.934	1.000	-				1	0.009	0.899	0.334
* Ase-ua4*	1	0.005	1.273	0.876	-				1	0.014	1.377	0.439
* Ase-ua5*	**1**	**0.004**	**0.986**	**0.048**	1	0.006	0.584	0.584	1	0.009	0.919	0.770
* Ase-ua6*	1	0.003	0.873	0.185	-				1	0.010	0.959	0.782
* Ase-ua7*	**1**	**0.004**	**1.150**	**0.010**	**1**	**0.009**	**0.995**	**< 0.001**	1	0.009	0.882	0.522
* Ase-ua8*	1	0.006	1.514	0.605	-				1	0.011	1.099	0.119
* Ase-ua9*	**1**	**0.003**	**0.899**	**0.011**	1	0.008	0.834	0.118	1	0.010	1.040	0.672
* Ase-ua11*	1	0.007	1.743	0.976	-				1	0.014	1.376	0.304
Age	**1**	0.004	0.937	0.971	1	0.015	1.532	0.518	1	0.014	1.353	0.916
Season	**1**	**0.007**	**1.966**	**0.001**	**1**	**0.020**	**2.145**	**< 0.001**	1	0.014	1.357	0.145
Sample Year	**5**	**0.038**	**1.986**	**< 0.001**	**6**	**0.083**	**1.455**	**< 0.001**	6	0.062	1.026	0.313
Sex	1	0.003	0.889	0.862	**1**	**0.014**	**1.481**	**< 0.001**	1	0.009	0.855	0.148
Days at 4°C	**1**	**0.010**	**2.543**	**0.009**	1	0.010	1.083	0.485	**1**	**0.013**	**1.322**	**0.012**
Time of day	**1**	**0.009**	**2.401**	**< 0.001**	**1**	**0.017**	**1.794**	**< 0.001**	1	0.014	1.428	0.123

16S GM composition was associated with the MHC-I variants, *Ase-ua 5*, *Ase-ua 7*, and *Ase-ua 9* (Table 2B, Fig. 1C-E), and also season, sample year, days at 4 °C, and time of day (Table 2B), but not genome-wide heterozygosity, age, and sex. Despite these significant associations between GM composition and MHC variation, the overall effect sizes were small across all MHC variables (R^2^ < 0.4%, Table 2, Fig. 1). Sample dispersion did not significantly differ with MHC-I variants, *Ase-ua 5*, *Ase-ua 7*, and *Ase-ua 9*, season, and sample year (Table S5).

#### Metagenomic taxonomic composition

**Fig. 2:**
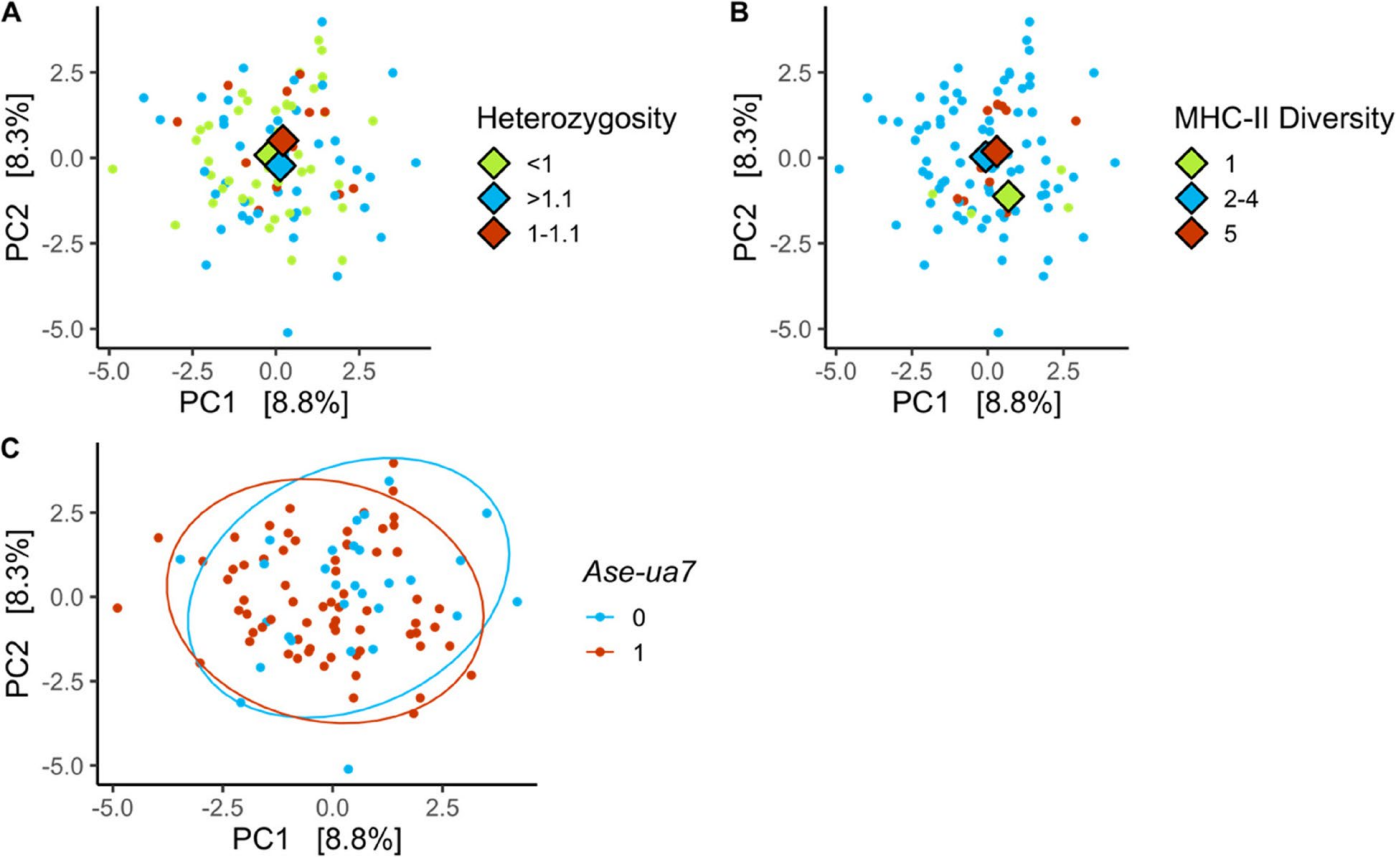
Principal Component Analyses (PCA) of gut microbiome metagenomic taxonomic compositional variation of Seychelles warbler faecal samples in relation to genome-wide heterozygosity (**A**), MHC-II diversity (**B**), and MHC-I allele *Ase-ua 7 *(**C**)*. N* = 99 from 57 birds. Large diamonds represent the group centroids. For clarity, samples were grouped into discrete categories for plotting. In plots **A** and **B**, the coloured points indicate low (green), middle (blue), and high (red) genome-wide heterozygosity (Heterozygosity) and MHC-II diversity, respectively. In plot **C**, blue = absence (0) and red = presence (1) of *Ase-ua 7*. Ellipses of each group are drawn around the points

Variation in GM metagenomic taxonomic composition was associated with genome-wide heterozygosity and MHC-II diversity, but not MHC-I diversity (Table 2A, Fig. 2A-B). Of the control variables, sex, sample year, and time of day were associated with metagenomics taxonomic composition (Table 2A), but age, season, and days at 4 °C were not.

When assessing MHC variants identified in the 16S analysis, GM metagenomic taxonomic composition was associated with the presence of MHC-I *Ase-ua 7* (Table 2B, Fig. 2C) but not *Ase-ua 5* and *Ase-ua 9*. Sex, season, sample year, and time of day were also associated with metagenomic taxonomic composition (Table 2B), but age and days at 4 °C were not. Despite significant differences in GM composition, the overall effect sizes were small across all MHC variables (R^2^ < 0.9%, Table 2, Fig. 2). Sample dispersion did not significantly differ with MHC-I variant, *Ase-ua 7*, season, and sample year (Table S5).

#### Metagenomics functional composition

**Fig. 3:**
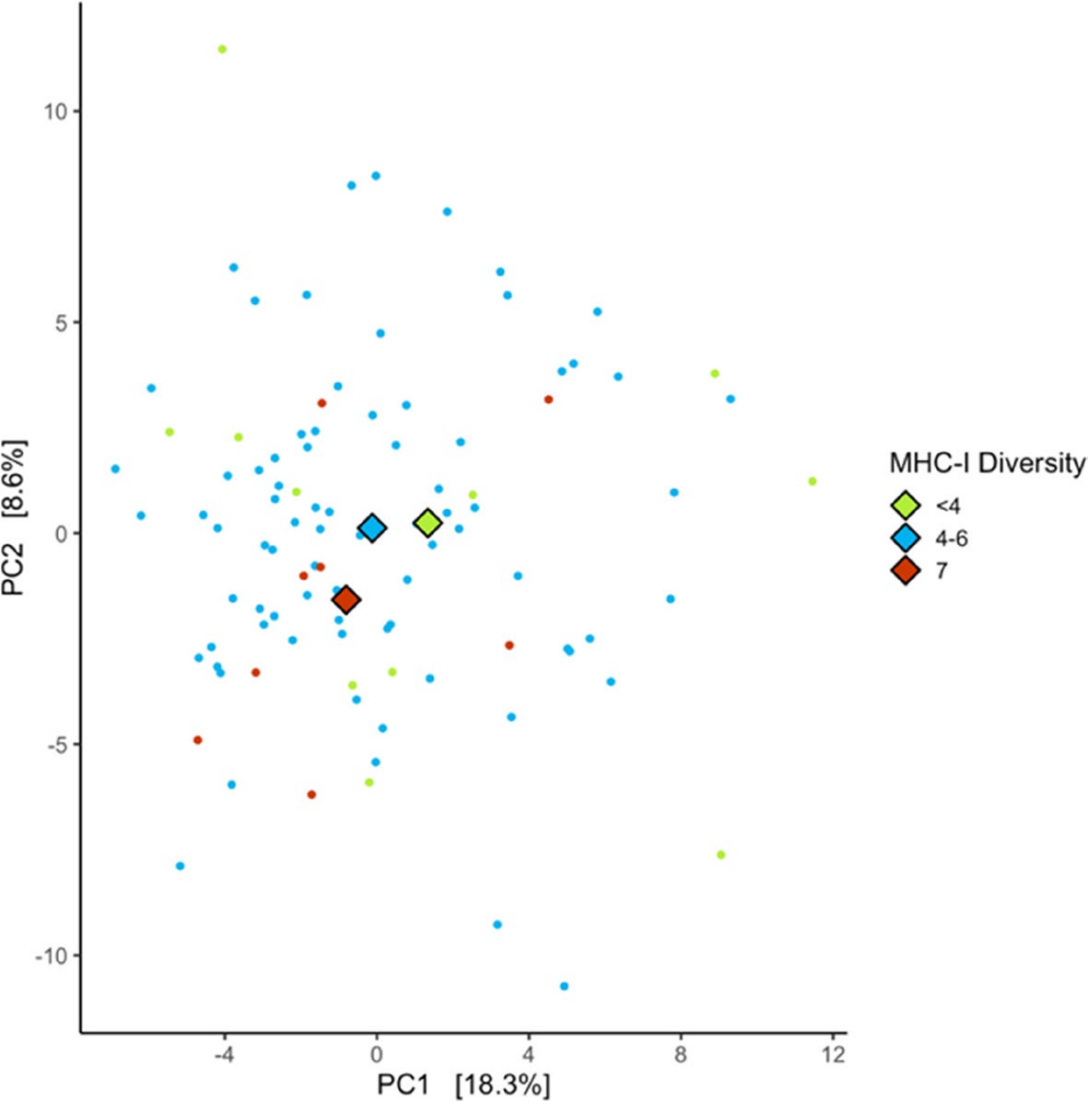
Principal Component Analyses (PCA) of gut microbiome compositional variation determined using metagenomics function with MHC-I diversity in the gut microbiome of Seychelles warblers (*n* = 99 from 57 birds). Large diamonds represent the group centroids. For clarity, samples were grouped into discrete categories for plotting. The coloured points represent the count < 4 (green), 4–6 (blue), and 7 (red) of MHC-I diversity. Principal components 1 and 2 explained 18.3% and 8.6% of gut microbiome structure, respectively

Functional GM composition was significantly associated with increasing individual MHC-I diversity (Table 2A, Fig. 3) and with days at 4 °C (Table 2A). However, genome-wide heterozygosity, MHC-II diversity and all other control variables (age, sex, season, sample year, and time of day) were not (Table 2A).

Functional GM composition was not significantly associated with any MHC alleles (Table 2B, Table S6). Functional GM composition was associated with days stored at 4 °C (Table 2B), but not with any other control variables (age, sex, season, sample year, time of day) (Table 2B). Despite significant differences in GM composition, the overall effect sizes were small across all MHC variables (R^2^ < 1.4%, Table 2, Fig. 3), but the MHC-I diversity effect size is the largest among GM compositional analyses. Sample dispersion did not significantly differ with season and sample year (Table S5).

### Differential abundance analysis

#### Differential abundance of metagenomic taxonomic species

**Fig. 4:**
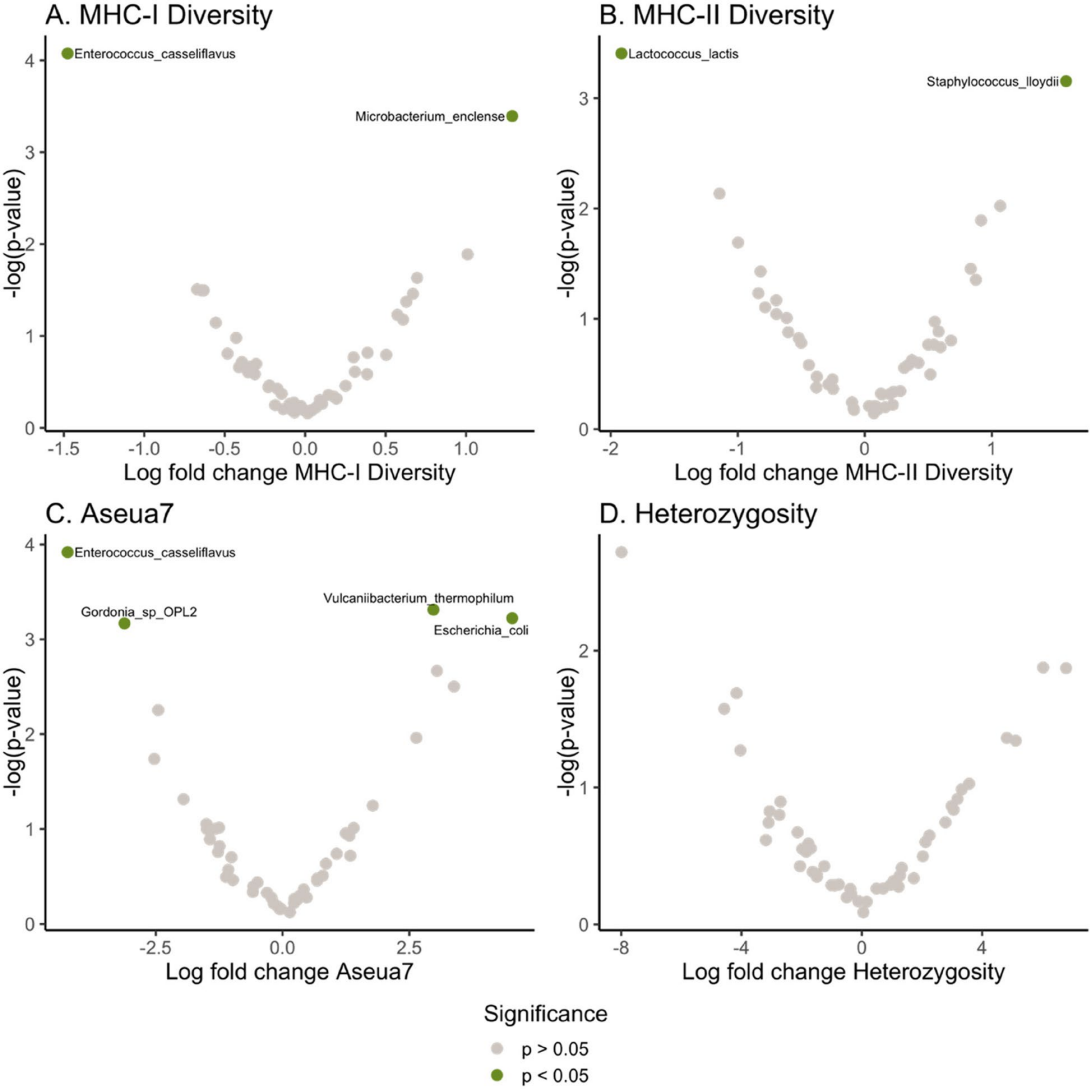
Differential abundance of metagenomically identified bacterial species in adult Seychelles warblers according to host genome-wide heterozygosity (**A**), MHC-I diversity (**B**), MHC-II diversity (**C**), and presence of MHC-I *Ase-ua 7* (*n* = 99 from 57 birds) (**D**). Points represent bacterial species and are coloured according to significance; green points (with species-level taxonomic annotations) are significantly differentially abundant (*p* < 0.05), and grey points are not

The abundance of some individual bacterial species (identified using metagenomics) varied in relation to MHC characteristics (Fig. 4AB); the abundance of *Enterococcus casselifavus* decreased, and *Microbacterium enclense* increased with increasing MHC-I diversity (Fig. 4A). The abundance of *Lactococcus lactis* decreased, and the abundance of *Staphylococcus lloydii* increased with increasing MHC-II diversity (Fig. 4B).

The abundances of four bacterial species were significantly associated with the presence/absence of the MHC-I allele *Ase-ua 7* (identified as associated with GM taxonomy composition), i.e. there was decreased prevalence of *Enterococcus casselifavus* and *Gordonia* sp OPL2, and an increased prevalence of *Escherichia coli* and *Vulcaniibacterium thermophilum,* when *Ase-ua 7* was present (Fig. 4C). The abundance of bacterial species was not significantly related to host genome-wide heterozygosity (Fig. 4D).

#### Differential abundance of metagenomic functional genes

**Fig. 5:**
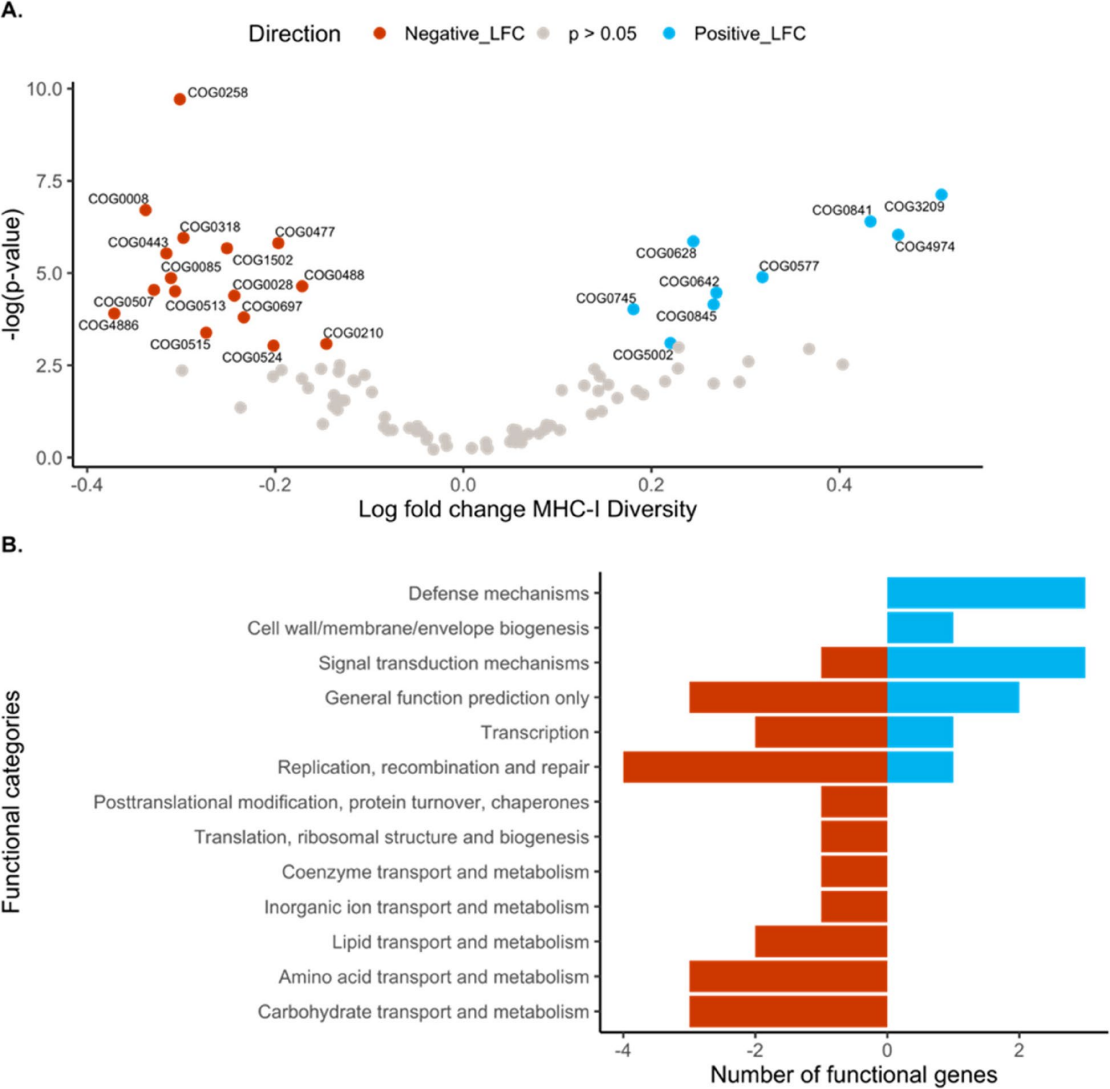
Variation in the abundance of bacterial functional genes (determined using eggNOG) in the gut microbiome of adult Seychelles warblers in relation to individual MHC-I diversity (*n* = 99 from 57 birds). **A** The results of an ALDEx2 differential abundance test showing the log fold change in abundance of each eggNOG gene annotation with increasing MHC-I diversity. Blue points are significantly (*p* < 0.05) more abundant, red points are significantly (*p* < 0.05) less abundant, and grey points do not differ significantly with MHC-I diversity. Labels on significant points represent eggNOG members. **B** Counts of functional genes per eggNOG category that demonstrated a significant positive (blue) or negative (red) log fold change with increasing MHC-I diversity, respectively

Abundances of 24 GM functional gene annotations differed significantly in relation to individual host MHC-I diversity (Table S7, Fig. 5A). In total, 9 GM genes increased in abundance and 15 genes decreased in abundance with increasing MHC-I diversity.

The 24 gene annotations were derived from 13 functional gene categories (defined by Cluster of Gene Orthologs (COG)). With increasing MHC-I diversity, two COG categories only increased in prevalence, four COG categories increased and decreased in prevalence, and seven COG categories only decreased in prevalence (Fig. 5B). Five of the seven COG categories that only decreased in prevalence are involved in bacterial metabolism. In addition, one GM functional gene annotation (COG1216) increased in prevalence with increasing genome-wide heterozygosity.

The KEGG orthologs and pathways of MHC-I diversity associated genes (Table S7) further support the findings, as core microbial functions decreased in prevalence; Carbohydrate metabolism (K01885, K01886, K01652, K06131, K01115), lipid metabolism (K00666), amino acid metabolism (K00852, K00847, K00874), transcription (K03043, K02335, K04799), translation (K08832, K15409), and replication and repair (K03581, K04043, K03283) (Table S7). In addition, MHC-I diversity was positively associated with KEGG pathways involved in environmental defence or stress adaptation (K02004, K18138, K09800, K04763; Table S7).

A direct test of trade-offs between functional categories of microbial defence and metabolism shows that as MHC-I diversity increases, the relative abundance of microbial defence functional category increases (Estimate = 0.32, *p* = 0.017, Table S8, Figure S2), whereas the relative abundance of microbial metabolism functional categories decreases (Estimate = −0.26, *p* = 0.001, Table S8, Figure S2).

Three GM functional gene annotations were differentially abundant with increasing MHC-II diversity: decreased in COG0318 (K00666 – fatty-acyl-CoA synthase), and increased in COG1609 (K02529 – LacI family transcriptional regulator, galactose operon repressor) and COG1653 (K02027 – multiple sugar transport system substrate-binding protein).

### Network analysis

#### Network analysis of metagenomic taxonomic species

**Fig. 6:**
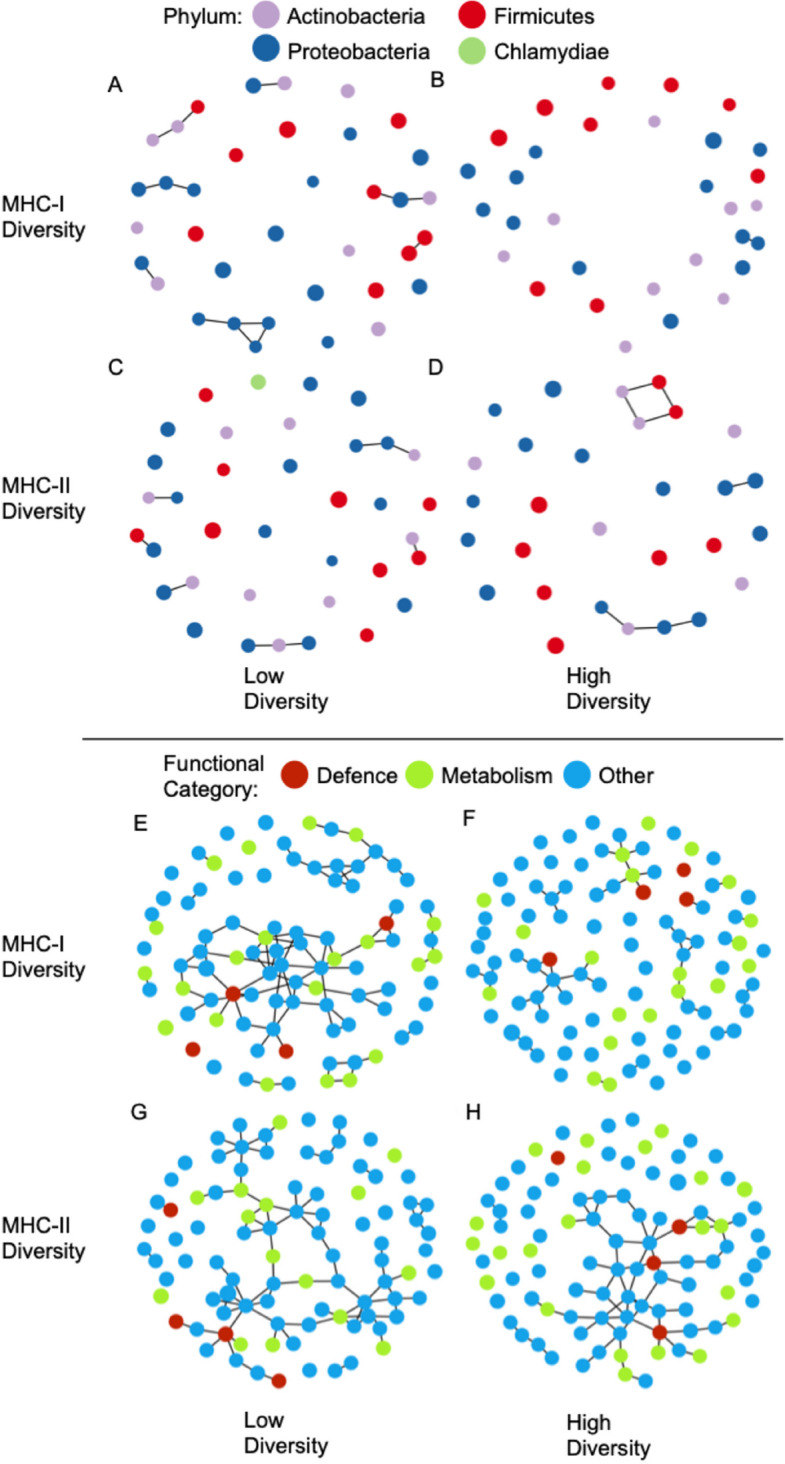
Network analysis between MHC diversity and the gut microbiome in adult Seychelles warblers (*n* = 99 from 57 birds). Each node represents (**A**-**D**) metagenomic taxonomic species or (**E**–**H**) metagenomic functional genes. MHC-I diversity (**ABEF**), MHC-II diversity (**CDGH**), low MHC diversity (**ACEG**), high MHC diversity (**BDFH**). Nodes (species/genes) are coloured by Phylum (**A**-**D**) and functional category (**E**–**H**). The size of the nodes is proportional to the mean abundance. Lines are edges (interaction between species/genes), connecting nodes that are linked

The metagenomic taxonomic species network had a higher number of connected nodes and edges in low MHC-I diversity (19 nodes, 13 edges) compared to high (2 nodes, 1 edge) MHC-I diversity individuals (Fig. 6AB). The average number of edges per connected node was also higher in low (mean = 1.4 edges per node) than in high MHC-I diversity (mean = 1.0 edges per node). Modularity was 0.82 in low MHC-I diversity, but zero in high MHC-I diversity due to only having one edge. The ratio of negative to positive edges in low MHC-I diversity was 0.3; there was only a single positive edge and no negative edges in MHC-I diversity. However, permutation tests show that edge density, modularity, and positive edge percentage of the metagenomic taxonomic species network did not significantly differ between low and high MHC-I diversity (Table S9).

In relation to MHC-II diversity, the metagenomic taxonomic species network had a higher number of connected nodes in low (14 nodes) than in high (10 nodes) MHC-II diversity individuals (Fig. 6CD). However, the number of edges did not differ between low (8 edges) and high (8 edges) MHC-II diversity (Fig. 6CD). The average number of edges per connected node was lower in low (mean = 1.1 edges per node) than in high (mean = 1.6 edges per node) MHC-II diversity. Modularity was also higher in low (0.81) than in high (0.59) MHC-II diversity. The ratio of negative to positive edges was 0.33 in low MHC-II diversity and was 0.14 in high MHC-II diversity. Permutation tests show that edge density, modularity, and positive edge percentage of the metagenomic taxonomic species network did not significantly differ between low and high MHC-II diversity (Table S9).

#### Network analysis of metagenomic functional genes

The metagenomic functional genes network had a higher number of connected nodes and edges in individuals with low MHC-I (76 nodes, 87 edges) than high MHC-I (56 nodes, 44 edges) diversity (Fig. 6EF). The average number of edges per connected node was also higher in low MHC-I (mean = 2.3 edges per node) than in high MHC-I diversity (mean = 1.6 edges per node). Modularity was lower in low MHC-I (0.72) than in high (0.85) MHC-I diversity. The ratio of negative to positive edges was 0.45 in low MHC-I diversity and 0.19 in high MHC-I diversity. Permutation tests show that the edge density of the metagenomic functional genes network was significantly higher in low compared to high MHC-I diversity (*p* = 0.04, Table S9). This shows that the metagenomically derived functional genes network is more fragmented in high MHC-I diversity individuals. Modularity and positive edge percentage of the metagenomic functional genes network did not significantly differ between low and high MHC-I diversity. (Table S9).

In relation to MHC-II diversity, the metagenomic functional genes network had a higher number of connected nodes and edges in low (74 nodes, 83 edges) than in high MHC-II diversity individuals (55 nodes, 62 edges) (Fig. 6GH). The average number of edges per connected node was very similar between low (mean = 2.2 edges per node) and high (mean = 2.3 edges per node) MHC-II diversity individuals. Modularity was also higher in low (0.76) than in high (0.65) MHC-II diversity individuals. The ratio of negative to positive edges was 0.26 in low MHC-II diversity and was 0.44 in high MHC-II diversity. However, permutation tests show that edge density, modularity, and positive edge percentage of the metagenomic functional genes network did not significantly differ between low and high MHC-II diversity (Table S9).

### MHC divergence vs. MHC diversity

In GM alpha diversity models, using MHC divergence resulted in a similar model fit (assessed via AIC) to models using the original MHC diversity measures (see supplementary; Table 11, Table S10). However, even in models where the fit was slightly improved by using MHC divergence (16S GM richness, metagenomic taxonomic richness, metagenomic functional richness, and metagenomic functional Shannon diversity), using MHC divergence had little impact on which specific variables were found to be significantly associated with GM diversity (i.e. they showed qualitatively the same results). Of the six alpha diversity models, only one model showed a significant change in variable significance; MHC-I divergence was significantly associated with metagenomic taxonomic richness, but not MHC-I diversity (Table S12).

In GM composition models, using MHC divergence did not improve model fit (AIC) and did not improve variance explained (R^2^) of any model compared to using the MHC diversity measure (Table 2, Table S11). Of the three GM composition models, only one model showed a significant change in variable significance; MHC-I divergence was not significantly associated with 16S GM composition, but MHC-I diversity was (Table S12).

## Discussion

Our study shows that in adult Seychelles warblers, there was no association between GM diversity (characterised by either 16S ASV or metagenomics) and MHC diversity, though one specific MHC variant (MHC-I allele *Ase-ua 11*) was associated with 16S GM richness but not metagenomic GM diversity. However, GM composition (16S and metagenomic-derived) was associated with MHC diversity. The 16S GM composition was associated with MHC-I and MHC-II diversity in a non-linear (quadratic function) manner, and with the MHC-I alleles *Ase-ua 5*, *Ase-ua 7*, and *Ase-ua 9*. The metagenomic taxonomic composition was associated with MHC-II diversity and the MHC-I allele *Ase-ua 7*. Furthermore, the overall functional composition of the GM (metagenomically derived) was associated with variation in MHC-I diversity. Two metagenomic bacterial species were differentially abundant with increasing MHC-I and MHC-II diversity, and four bacterial species differed in relation to the presence/absence of MHC-I allele *Ase-ua 7*. Furthermore, 24 functional genes differed in abundance with increasing MHC-I diversity (this corresponded to increases in microbial defence mechanisms and decreases in microbial metabolism) and three with increasing MHC-II diversity. Network analysis showed that high (compared to low) MHC-I diversity was also associated with greater fragmentation in functional GM structure.

Consistent with previous findings on the Seychelles warbler, we found that MHC diversity (either class-I or class-II) was not significantly associated with GM alpha diversity [21, 110]. This contradicts results found in other wild populations [6, 41, 58, 101]. For example, in giant salamanders (*Cryptobranchus alleganiensis bishopi* and *C. a. alleganiensis*) and sticklebacks (*Gasterosteus aculeatus*), increasing MHC allelic diversity is correlated with increases and decreases in overall skin microbiome diversity, respectively [6, 41]. Given that the Seychelles warblers had a recent population bottleneck (< 50 individuals in the 1960 s, [95]), the MHC is less diverse than in most similar species [88], and may therefore have a reduced impact on GM diversity. However, even so, considerable MHC variation remains in the Cousin Island warbler population [88] and the most functionally divergent MHC alleles were likely retained, as such alleles are more likely to differ in antigen-binding properties and thus confer broader immunological protection [72, 88]. This may also explain why replacing MHC diversity with MHC divergence had little effect on our overall results as there is little difference between these metrics in this species. Thus, we conclude that MHC and GM diversity associations may be host-species dependent and functionally dependent regardless of overall MHC diversity/divergence levels.

In contrast to the GM diversity results, shifts in GM composition (both 16S and metagenomic) were associated with MHC characteristics. An association between MHC-II diversity and GM taxonomic composition, as seen in our study, has been shown multiple times in captive and wild systems (see review [90]). In our study, only two bacterial species differed in abundance among individuals with different levels of MHC diversity (increased *Staphylococcus loydii* and decreased *Lactococcus lactis*)*.* There is evidence of *Lactococcus lactis* causing detrimental infections in avian species [36], but it has also been used as a probiotic for broiler chickens (*Gallus gallus domesticus*) [9, 69]. *Staphylococcus loydii* hasn’t been observed to cause infections in any species. These results may suggest that in the Seychelles warblers, increasing MHC-II diversity may suppress pathogenic species. In addition, the MHC-I allele *Ase-ua 7* was significantly correlated with differences in GM composition, consistent with previous work on this population [21]. *Ase-ua 7* was associated with decreases in *Enterococcus casselifavus* and *Gordonia* sp OPL2, (gram-positive bacteria), but increased prevalence of the gram-negative bacteria, *Escherichia coli* and *Vulcaniibacterium thermophilum*, [57, 62, 70, 116]. The World Health Organization (WHO) priority list of pathogens primarily consists of Gram-negative bacteria [10]. Therefore, we speculate that the effect of *Ase-ua 7* on GM composition could be based on microbial cell wall structure and could be important in controlling pathogens. Indeed, during inflammation, MHC expression was upregulated in the small intestinal stem cells of humans and mice (*Mus musculus*) [42].

In our study, the effects of MHC-II diversity and the MHC-I allele *Ase-ua 7* on taxonomic GM are relatively small (two and four, out of 49, core species were differentially abundant, respectively). Such limited effects have also been found in the few previous similar studies, with the MHC variations influencing only a very small number of bacterial species rather than the overall composition [6, 68, 110]. This small taxonomic effect could explain why both MHC-II diversity and MHC-I allele *Ase-ua 7* were not significantly associated with metagenomic function (see below). As only a few microbial species are influenced, other microbes may replace their function through functional redundancy [64, 112]. Moreover, network analyses of both taxonomic and functional GM revealed that MHC-II diversity was not associated with greater fragmentation, modularity, and a negative-to-positive interaction ratio; these differences were more pronounced for MHC-I diversity. This further supports the conclusion that MHC-II diversity has a relatively small effect on the gut microbiome.

In contrast, the diversity of MHC-I alleles (but not class-II) did appear to influence GM functional differences (determined metagenomically), with higher MHC-I diversity leading to the presence of an increased number of microbial defence genes, whilst decreasing the number of metabolism-related genes. This is further shown in network analysis, where functional microbial networks appear more fragmented in individuals with high MHC-I diversity, indicating reduced microbial interactions. These patterns may reflect increased microbial competition, fewer metabolic interactions among microbes, or immune-mediated disruption under high MHC-I diversity. Trade-offs between defence and growth are common (although not ubiquitous) in microbial species [27, 63]. Our results suggest that these microbial trade-offs can also amount to costs for the host, whereby control of the microbiome (via the immune system) can result in a reduction in the GM’s metabolic potential. Presumably, these costs may be outweighed by the benefits of eliminating pathogens and maintaining a healthy microbiome [34, 67]. Quantifying the relative costs and benefits of maintaining a dynamic microbiome remains largely unexplored but is essential for understanding how host-microbiome interactions and host control mechanisms (including the immune system) evolve [34, 67, 105].

Overall, our study indicates that MHC-I, not MHC-II, plays a greater role in shaping the GM, both taxonomically and functionally. This is consistent with previous findings in the Seychelles warbler [21, 110], as well as in the reddish-gray mouse lemur (*Microcebus griseorufus*) [68]. MHC-I molecules encode receptors that typically act intracellularly, while MHC-II receptors interact extracellularly. Thus, intuitively the class-II receptors should have greater interaction with and influence over gut microbes [89]. However, the mechanisms by which MHC-I alleles and diversity influence GM function remain unclear [90]. MHC-I receptors can be triggered by bacteriophages [2], which play an important role in shaping bacterial composition [45]. Future work on host MHC and the gut virome in the Seychelles warbler could help to elucidate the mechanisms by which MHC-I influences GM composition.

It is also possible that MHC-I indirectly impacts the gut microbiome via effects on host health. In the Seychelles warblers, MHC-I diversity has been positively correlated with both survival and reproductive success [12, 87, 88]. In the current study, we also found that MHC-I diversity was associated with GM metagenomic function. It is plausible that MHC-I diversity affects GM functionality by controlling mutualistic bacteria, indirectly influencing host survival. However, MHC-I variation could affect other components of the host’s overall health, indirectly leading to differences in GM function. Consequently, multiple mechanisms may be involved in the relationship between MHC-I diversity and individual differences in the GM.

An individual’s sex and genome-wide heterozygosity, independent of MHC variation, were also associated with shifts in GM metagenomic species. However, these changes were not detectable in terms of 16S ASVs and metagenomics function, which indicates that the changes are species/strain specific and do not influence microbial function. This result is reinforced by the fact that no common species were differentially abundant with increasing levels of genome-wide heterozygosity. Various other variables including sample year, season, and time of day, were also predictors of 16S ASVs and metagenomic species, but not metagenomic function. The fact that so many factors affect the species present in the GM, but not the overall GM functionality is likely attributed to functional redundancy, as the types of environmental microbes change with time, the overall GM function is replaced by other microbes (potentially due to changes in diet), thereby preserving the overall GM function [64, 112].

Interestingly, the number of days stored at 4 °C was associated with shifts in metagenomics function but not 16S ASVs and metagenomic species. Larger DNA fragments degrade quicker in storage, and these larger DNA fragments are required for accurate gene recognition and gene annotation, as these steps require a start codon followed by an open reading frame [47]. Whereas smaller DNA fragments may still be sufficient for accurate taxonomic assignment of 16S ASVs and metagenomic species, as specific marker genes are typically used for taxonomic annotations [4, 74].

One limitation of our study is that it is purely correlative, and we are unable to validate our findings experimentally. Nonetheless, our findings in both datasets (16S and metagenomic methods) are comparable despite the smaller sample size in the metagenomic work. Future experimental work in more amenable study systems could investigate potential pathways by which host MHC-I diversity and alleles may affect the GM, for example by introducing immune-triggering bacteriophages and measuring their impact on the gut bacteriophage and bacteria community. [35, 104]. However, such experiments are likely lab-based and the GM would be radically altered [102], hence the generalisation to natural populations would be limited. A further limitation of our study is that it lacks gene expression data for the MHC and the GM. Therefore, the functional relevance is only on the DNA level, reflecting potential rather than actual function of the MHC and GM. However, in the bottlenecked Seychelles warbler population, the few remaining MHC alleles are highly divergent, preventing further reduction into functional supertypes [21, 88, 113]. Future research could use transcriptomics and proteomics to quantify both MHC and GM function. The lack of expression data could plausibly explain the modest effect sizes in this study as a locus could be present but not expressing. In addition, although multiple studies have found that MHC is associated with the microbiome [6, 41, 58, 101], the host regulation of the GM may be more directly shaped by innate immunity, such as by toll-like receptors, NOD-like receptors or defensins [105]. However, a previous study in the Seychelles warbler found no associations between toll-like receptor 3 (variation at which influences host survival in this species) and the GM alpha diversity or composition [21]. Therefore, while the MHC may only explain a limited amount of variation in the GM (as shown in this study and [21, 110]), this variation may still be important.

Despite reporting several associations between MHC characteristics and the GM, our study is limited to only assessing genome-wide heterozygosity and the specific MHC candidate alleles we had already screened for. A genome-wide association study on the host and its GM could reveal more loci, and potentially more nuanced or polygenic effects, by accounting for multiple genes concurrently [114]. However, this requires whole-genome data on many individual birds, in combination with metagenomic sequencing of their GM, which would be very costly and labour-intensive [56, 115]. Leveraging a sequencing approach that targets specific individuals and utilises recent technological advances, such as the imputation of low-coverage samples, could be a feasible way to conduct such studies [115]. In the same vein, while heterozygosity measured using 30 neutral microsatellite loci does reflect genome-wide heterozygosity/inbreeding in this species [95], whole-genome sequencing of individuals would be a more accurate measure. It would enable us to determine runs of homozygosity, and thus provide the resolution needed for a powerful investigation of the effect of host inbreeding on the GM [17].

In conclusion, our study suggests that both MHC class I and II influence an individual’s GM in the Seychelles warbler, but that despite being an intracellular receptor, MHC-I has a greater influence on GM composition, than MHC-II. We also found that MHC-I allele *Ase-ua 7* changes the GM taxonomic composition, while MHC-I diversity alters GM function. These results may explain previous findings that MHC-I diversity is positively correlated with fitness in this population [12, 87], potentially as a result of inducing changes in the GM functionality. However, this could be an indirect effect, rather than the GM actively contributing to increased fitness. Various pathways are involved in regulating the immune system, underscoring the need for host and gut transcriptomics and metabolomic data to enable mechanistic investigation of immunogenetics and the GM [24, 90].

## Supplementary Information

Additional file 1: Supplementary materials.

## Data Availability

All 16S sequencing data used in this study can be accessed from European Nucleotide Archive (ENA) database under the study accession numbers PRJEB45408 (samples taken in 2017 and 2018) and PRJEB47095 (samples taken in 2019 and 2020) and PRJEB67634 (samples taken in 2021 and 2022). All shotgun metagenomics data used in this study can be accessed from ENA database under the study accession number PRJEB81709.
