# Supplementary material for: Host immunogenetic variation and gut microbiome functionality in a wild vertebrate population
Source: Microbiome. 2026 Mar 12;14:78. doi: 10.1186/s40168-026-02341-9 (PMC12980968; doi:10.1186/s40168-026-02341-9)
Supplement: Supplementary file 2 — Additional file 1: Supplementary materials. [file 40168_2026_2341_MOESM1_ESM.docx]

# Supplementary materials for:

# Host immunogenetic variation and gut microbiome functionality in a wild vertebrate population

Chuen Zhang Lee^1,2*^, Sarah F. Worsley^1^, Charli S. Davies^1^, Jan Komdeur^3^, Falk Hildebrand^1,4,5^, Hannah L. Dugdale^3^, David S. Richardson^1,6*^

^1^ School of Biological Sciences, University of East Anglia, Norwich, Norfolk, UK

^2^ Centre for Microbial Interactions, Norwich Research Park, Norwich, Norfolk, UK

^3^ Groningen Institute for Evolutionary Life Sciences (GELIFES), University of Groningen, Groningen, The Netherlands

^4^ Quadram Institute Bioscience, Norwich Research Park, Norfolk, UK

^5^ Earlham Institute, Norwich Research Park, Norfolk, UK

^6^ Nature Seychelles, Roche Caiman, Mahé, Republic of Seychelles

*Correspondence: david.richardson@uea.ac.uk, chuen.lee@uea.ac.uk

**Supplementary tables**

Table S1. The relationship between gut microbiome alpha diversity (Shannon) and variation in host (A) MHC diversity and (B) the presence/absence of specific MHC alleles in Seychelles warblers. Linear mixed models were used for all models. N = 253 samples from 149 individuals in 16S ASV diversity and N = 99 samples from 57 individuals in metagenomic taxonomy diversity and functional diversity. Reference categories for categorical variables were as follows: Female (sex), winter (season), 2017 (Sample year), and absent (0) in all MHC alleles. Significant (P < 0.05) variables are shown in bold.

| Model | Predictor | 16S ASV Shannon | | | | | Metagenomics Taxonomy Shannon | | | | | Metagenomics Functional Shannon | | | | |
| --- | --- | --- | --- | --- | --- | --- | --- | --- | --- | --- | --- | --- | --- | --- | --- | --- |
| A) MHC Diversity |  | Est | SE | df | t | P | Est | SE | df | t | P | Est | SE | df | t | P |
|  | (Intercept) | **3.256** | **0.609** | **151.658** | **5.344** | **< 0.001** | 0.707 | 0.843 | 51.987 | 0.838 | 0.406 | 810.308 | 458.228 | 63.471 | 1.768 | 0.082 |
|  | Heterozygosity | 0.035 | 0.400 | 127.858 | 0.087 | 0.931 | 0.549 | 0.529 | 43.259 | 1.039 | 0.304 | 84.219 | 284.505 | 56.648 | 0.296 | 0.768 |
|  | MHC-I Diversity | 0.043 | 0.059 | 126.354 | 0.726 | 0.470 | 0.056 | 0.079 | 39.364 | 0.705 | 0.485 | 18.886 | 41.001 | 47.343 | 0.461 | 0.647 |
|  | MHC-II Diversity | -0.038 | 0.085 | 107.453 | -0.444 | 0.658 | -0.106 | 0.101 | 36.388 | -1.043 | 0.304 | -8.708 | 54.004 | 49.176 | -0.161 | 0.873 |
|  | Age | -0.026 | 0.034 | 167.775 | -0.742 | 0.459 | -0.015 | 0.040 | 54.211 | -0.383 | 0.703 | -41.418 | 22.222 | 64.476 | -1.864 | 0.067 |
|  | Season | -0.152 | 0.218 | 232.175 | -0.698 | 0.486 | 0.002 | 0.246 | 81.629 | 0.007 | 0.994 | 147.236 | 147.488 | 69.650 | 0.998 | 0.322 |
|  | Sex | -0.276 | 0.164 | 116.709 | -1.677 | 0.096 | 0.204 | 0.217 | 37.363 | 0.941 | 0.353 | 4.794 | 113.824 | 47.563 | 0.042 | 0.967 |
|  | Days in fridge | -0.084 | 0.177 | 237.601 | -0.475 | 0.635 | -0.235 | 0.191 | 65.090 | -1.232 | 0.222 | 30.756 | 124.123 | 75.900 | 0.248 | 0.805 |
|  | Catch Time | -0.023 | 0.159 | 238.584 | -0.145 | 0.885 | 0.112 | 0.188 | 74.829 | 0.594 | 0.554 | 79.915 | 117.418 | 78.638 | 0.681 | 0.498 |
|  | SampleYear2018 | 0.168 | 0.237 | 232.771 | 0.710 | 0.478 | 0.216 | 0.299 | 81.766 | 0.721 | 0.473 | 95.562 | 172.637 | 76.217 | 0.554 | 0.582 |
|  | SampleYear2019 | -0.092 | 0.306 | 238.742 | -0.301 | 0.764 | -0.188 | 0.397 | 79.019 | -0.473 | 0.637 | 90.988 | 238.156 | 78.404 | 0.382 | 0.704 |
|  | SampleYear2020 | 0.488 | 0.393 | 232.913 | 1.243 | 0.215 | 0.353 | 0.482 | 75.198 | 0.733 | 0.466 | -75.215 | 292.792 | 78.329 | -0.257 | 0.798 |
|  | SampleYear2021 | -0.179 | 0.300 | 238.987 | -0.597 | 0.551 | 0.074 | 0.375 | 67.298 | 0.198 | 0.844 | 84.213 | 237.966 | 75.655 | 0.354 | 0.724 |
|  | SampleYear2022 | 0.114 | 0.292 | 232.157 | 0.391 | 0.696 | 0.270 | 0.347 | 81.205 | 0.778 | 0.439 | 285.653 | 202.309 | 76.433 | 1.412 | 0.162 |
|  | SampleYear2023 |  |  |  |  |  | 0.425 | 0.391 | 81.858 | 1.087 | 0.280 | 133.373 | 243.410 | 78.918 | 0.548 | 0.585 |
| B) Presence/absence of MHC alleles | (Intercept) | **3.770** | **0.643** | **133.977** | **5.864** | **< 0.001** | **1.262** | **0.314** | **82.917** | **4.018** | **< 0.001** | 910.63 | 503.20 | 44.89 | 1.81 | 0.077 |
|  | Heterozygosity | **0.119** | **0.411** | **111.652** | **0.291** | **0.772** |  |  |  |  |  | 264.30 | 321.88 | 43.24 | 0.82 | 0.416 |
|  | *Ase-dab3* | 0.306 | 0.289 | 142.300 | 1.061 | 0.290 |  |  |  |  |  | 151.44 | 225.26 | 30.39 | 0.67 | 0.506 |
|  | *Ase-dab4* | -0.288 | 0.301 | 126.639 | -0.958 | 0.340 |  |  |  |  |  | -324.40 | 241.83 | 33.75 | -1.34 | 0.189 |
|  | *Ase-dab5* | -0.031 | 0.304 | 146.426 | -0.103 | 0.918 |  |  |  |  |  | 216.57 | 271.27 | 34.33 | 0.80 | 0.43 |
|  | *Ase-ua1* | 0.153 | 0.360 | 123.331 | 0.424 | 0.672 |  |  |  |  |  | 172.81 | 263.46 | 35.70 | 0.66 | 0.516 |
|  | *Ase-ua3* | -0.332 | 0.370 | 122.950 | -0.898 | 0.371 |  |  |  |  |  | 168.94 | 284.69 | 29.39 | 0.59 | 0.557 |
|  | *Ase-ua4* | -0.277 | 0.276 | 107.669 | -1.005 | 0.317 |  |  |  |  |  | -61.61 | 245.46 | 34.87 | -0.25 | 0.803 |
|  | *Ase-ua5* | -0.240 | 0.348 | 133.132 | -0.690 | 0.491 |  |  |  |  |  | 131.65 | 294.06 | 35.24 | 0.45 | 0.657 |
|  | *Ase-ua6* | -0.004 | 0.342 | 140.968 | -0.012 | 0.991 |  |  |  |  |  | -391.22 | 265.32 | 29.45 | -1.48 | 0.151 |
|  | *Ase-ua7* | -0.272 | 0.401 | 118.988 | -0.679 | 0.499 |  |  |  |  |  | -258.03 | 268.05 | 30.80 | -0.96 | 0.343 |
|  | *Ase-ua8* | 0.004 | 0.284 | 127.433 | 0.013 | 0.990 |  |  |  |  |  | 0.82 | 224.58 | 34.52 | 0.00 | 0.997 |
|  | *Ase-ua9* | -0.188 | 0.337 | 115.587 | -0.557 | 0.579 |  |  |  |  |  | 38.05 | 298.07 | 42.97 | 0.13 | 0.899 |
|  | *Ase-ua11* | 0.494 | 0.363 | 115.143 | 1.359 | 0.177 | 0.199 | 0.203 | 41.077 | 0.977 | 0.334 | 80.78 | 278.76 | 34.89 | 0.29 | 0.774 |
|  | Age | -0.027 | 0.036 | 170.485 | -0.753 | 0.453 | -0.034 | 0.039 | 59.801 | -0.857 | 0.395 | -47.33 | 28.49 | 55.35 | -1.66 | 0.102 |
|  | Season | -0.208 | 0.222 | 223.919 | -0.939 | 0.349 | -0.010 | 0.243 | 83.916 | -0.040 | 0.968 | 213.15 | 161.05 | 61.82 | 1.32 | 0.191 |
|  | Sex | -0.268 | 0.171 | 114.037 | -1.565 | 0.120 | 0.102 | 0.210 | 42.108 | 0.487 | 0.628 | -19.91 | 134.26 | 41.34 | -0.15 | 0.883 |
|  | Days in fridge | -0.155 | 0.179 | 228.097 | -0.866 | 0.387 | -0.264 | 0.187 | 67.914 | -1.406 | 0.164 | 55.76 | 129.25 | 67.24 | 0.43 | 0.668 |
|  | Catch Time | 0.045 | 0.161 | 228.854 | 0.279 | 0.780 | 0.123 | 0.187 | 75.298 | 0.658 | 0.513 | 20.28 | 127.00 | 68.28 | 0.16 | 0.874 |
|  | SampleYear2018 | 0.147 | 0.238 | 226.562 | 0.615 | 0.539 | 0.242 | 0.299 | 83.252 | 0.810 | 0.420 | 18.69 | 190.33 | 64.41 | 0.10 | 0.922 |
|  | SampleYear2019 | -0.099 | 0.306 | 228.897 | -0.325 | 0.746 | -0.173 | 0.392 | 80.974 | -0.440 | 0.661 | -59.70 | 251.10 | 65.67 | -0.24 | 0.813 |
|  | SampleYear2020 | 0.479 | 0.398 | 226.558 | 1.203 | 0.230 | 0.331 | 0.478 | 76.291 | 0.693 | 0.491 | -101.98 | 324.61 | 68.96 | -0.31 | 0.754 |
|  | SampleYear2021 | -0.164 | 0.304 | 228.678 | -0.538 | 0.591 | 0.205 | 0.370 | 69.594 | 0.553 | 0.582 | -22.35 | 256.54 | 67.58 | -0.09 | 0.931 |
|  | SampleYear2022 | 0.087 | 0.300 | 218.106 | 0.291 | 0.771 | 0.333 | 0.346 | 83.825 | 0.964 | 0.338 | 191.29 | 230.43 | 64.46 | 0.83 | 0.41 |
|  | SampleYear2023 |  |  |  |  |  | 0.514 | 0.388 | 83.636 | 1.325 | 0.189 | 52.66 | 272.47 | 67.44 | 0.19 | 0.847 |

Table S2. The standardised effect sizes (partial R^2^) of the relationship between gut microbiome alpha diversity (richness) and variation in host (A) Major histocompatibility complex (MHC) diversity and (B) the presence/absence of specific MHC alleles in Seychelles warblers (Table 1 in main text).

| Model | Predictor | 16S ASV diversity | | | Metagenomics taxonomic diversity | | | Metagenomics functional diversity | | |  |
| --- | --- | --- | --- | --- | --- | --- | --- | --- | --- | --- | --- |
|  |  | Rsq | upper.CL | lower.CL | Rsq | upper.CL | lower.CL | Rsq | upper.CL | lower.CL | |
| A) MHC Diversity | Model | 0.088 | 0.202 | 0.067 | 0.128 | 0.357 | 0.114 | 0.169 | 0.397 | 0.140 | |
|  | Heterozygosity | 0.003 | 0.032 | <0.001 | 0.011 | 0.089 | <0.001 | 0.003 | 0.066 | <0.001 | |
|  | MHC-I Diversity | 0.006 | 0.039 | <0.001 | <0.001 | 0.052 | <0.001 | 0.034 | 0.139 | <0.001 | |
|  | MHC-II Diversity | <0.001 | 0.021 | <0.001 | 0.028 | 0.125 | <0.001 | 0.001 | 0.058 | <0.001 | |
|  | Age | 0.007 | 0.043 | <0.001 | 0.013 | 0.094 | <0.001 | 0.049 | 0.165 | 0.001 | |
|  | Season (summer) | <0.001 | 0.021 | <0.001 | 0.008 | 0.081 | <0.001 | 0.001 | 0.057 | <0.001 | |
|  | Sex (male) | 0.024 | 0.075 | 0.001 | 0.006 | 0.074 | <0.001 | 0.001 | 0.060 | <0.001 | |
|  | Days at 4°C | <0.001 | 0.020 | <0.001 | <0.001 | 0.052 | <0.001 | 0.004 | 0.069 | <0.001 | |
|  | Time of day | <0.001 | 0.020 | <0.001 | 0.024 | 0.119 | <0.001 | 0.003 | 0.068 | <0.001 | |
|  | Sample Year (2018) | 0.036 | 0.110 | 0.015 | 0.043 | 0.207 | 0.024 | 0.064 | 0.236 | 0.032 | |
| B) Presence/absence of MHC alleles | Model | 0.130 | 0.273 | 0.122 | 0.090 | 0.309 | 0.082 | 0.223 | 0.483 | 0.228 | |
|  | Heterozygosity | <0.001 | 0.022 | <0.001 |  |  |  | 0.001 | 0.059 | <0.001 | |
|  | *Ase-dab3* | 0.013 | 0.055 | <0.001 |  |  |  | 0.038 | 0.146 | <0.001 | |
|  | *Ase-dab4* | 0.011 | 0.050 | <0.001 |  |  |  | 0.011 | 0.092 | <0.001 | |
|  | *Ase-dab5* | 0.004 | 0.036 | <0.001 |  |  |  | 0.000 | 0.054 | <0.001 | |
|  | *Ase-ua1* | 0.002 | 0.027 | <0.001 |  |  |  | 0.001 | 0.056 | <0.001 | |
|  | *Ase-ua3* | 0.001 | 0.025 | <0.001 |  |  |  | 0.000 | 0.054 | <0.001 | |
|  | *Ase-ua4* | 0.006 | 0.041 | <0.001 |  |  |  | 0.006 | 0.077 | <0.001 | |
|  | *Ase-ua5* | 0.001 | 0.024 | <0.001 |  |  |  | 0.000 | 0.054 | <0.001 | |
|  | *Ase-ua6* | 0.004 | 0.034 | <0.001 |  |  |  | 0.005 | 0.075 | <0.001 | |
|  | *Ase-ua7* | 0.002 | 0.029 | <0.001 |  |  |  | 0.004 | 0.071 | <0.001 | |
|  | *Ase-ua8* | <0.001 | 0.021 | <0.001 |  |  |  | 0.007 | 0.080 | <0.001 | |
|  | *Ase-ua9* | 0.001 | 0.023 | <0.001 |  |  |  | 0.013 | 0.096 | <0.001 | |
|  | *Ase-ua11* | 0.015 | 0.059 | <0.001 | 0.002 | 0.060 | <0.001 | 0.035 | 0.141 | <0.001 | |
|  | Age | 0.010 | 0.049 | <0.001 | 0.026 | 0.121 | <0.001 | 0.033 | 0.137 | <0.001 | |
|  | Season | <0.001 | 0.021 | <0.001 | 0.004 | 0.068 | <0.001 | 0.001 | 0.057 | <0.001 | |
|  | Sex | 0.028 | 0.081 | 0.002 | 0.001 | 0.056 | <0.001 | 0.006 | 0.076 | <0.001 | |
|  | Days at 4°C | <0.001 | 0.022 | <0.001 | 0.001 | 0.057 | <0.001 | 0.004 | 0.071 | <0.001 | |
|  | Time of day | 0.003 | 0.030 | <0.001 | 0.028 | 0.126 | <0.001 | 0.003 | 0.065 | <0.001 | |
|  | Sample Year | 0.031 | 0.103 | 0.012 | 0.037 | 0.198 | 0.022 | 0.072 | 0.246 | 0.036 | |

Table S3. The standardised effect sizes (partial R^2^) of the relationship between gut microbiome alpha diversity (richness) and variation in host (A) Major histocompatibility complex (MHC) diversity and (B) the presence/absence of specific MHC alleles in Seychelles warblers (Table S1).

| Model | Predictor | 16S ASV diversity | | | Metagenomics taxonomic diversity | | | Metagenomics functional diversity | | |
| --- | --- | --- | --- | --- | --- | --- | --- | --- | --- | --- |
|  |  | Rsq | upper.CL | lower.CL | Rsq | upper.CL | lower.CL | Rsq | upper.CL | lower.CL |
| A) MHC Diversity | Model | 0.046 | 0.154 | 0.041 | 0.103 | 0.335 | 0.100 | 0.103 | 0.339 | 0.101 |
|  | Heterozygosity | <0.001 | 0.020 | <0.001 | 0.014 | 0.096 | <0.001 | 0.001 | 0.058 | <0.001 |
|  | MHC-I Diversity | 0.002 | 0.029 | <0.001 | 0.007 | 0.077 | <0.001 | 0.002 | 0.064 | <0.001 |
|  | MHC-II Diversity | 0.001 | 0.024 | <0.001 | 0.015 | 0.099 | <0.001 | <0.001 | 0.055 | <0.001 |
|  | Age | 0.002 | 0.029 | <0.001 | 0.002 | 0.060 | <0.001 | 0.037 | 0.145 | <0.001 |
|  | Season (summer) | 0.002 | 0.028 | <0.001 | <0.001 | 0.052 | <0.001 | 0.011 | 0.091 | <0.001 |
|  | Sex (male) | 0.012 | 0.052 | <0.001 | 0.012 | 0.092 | <0.001 | <0.001 | 0.054 | <0.001 |
|  | Days at 4°C | 0.001 | 0.024 | <0.001 | 0.013 | 0.094 | <0.001 | 0.001 | 0.057 | <0.001 |
|  | Time of day | <0.001 | 0.020 | <0.001 | 0.003 | 0.066 | <0.001 | 0.005 | 0.074 | <0.001 |
|  | Sample Year (2018) | 0.015 | 0.078 | 0.007 | 0.040 | 0.201 | 0.023 | 0.034 | 0.197 | 0.022 |
| B) Presence/absence of MHC alleles | Model | 0.081 | 0.226 | 0.089 | 0.078 | 0.297 | 0.075 | 0.150 | 0.434 | 0.185 |
|  | Heterozygosity | <0.001 | 0.022 | <0.001 |  |  |  | 0.008 | 0.082 | <0.001 |
|  | *Ase-dab3* | 0.005 | 0.036 | <0.001 |  |  |  | 0.005 | 0.075 | <0.001 |
|  | *Ase-dab4* | 0.004 | 0.034 | <0.001 |  |  |  | 0.021 | 0.114 | <0.001 |
|  | *Ase-dab5* | <0.001 | 0.020 | <0.001 |  |  |  | 0.007 | 0.081 | <0.001 |
|  | *Ase-ua1* | 0.001 | 0.023 | <0.001 |  |  |  | 0.005 | 0.074 | <0.001 |
|  | *Ase-ua3* | 0.003 | 0.033 | <0.001 |  |  |  | 0.004 | 0.071 | <0.001 |
|  | *Ase-ua4* | 0.004 | 0.035 | <0.001 |  |  |  | 0.001 | 0.057 | <0.001 |
|  | *Ase-ua5* | 0.002 | 0.028 | <0.001 |  |  |  | 0.002 | 0.064 | <0.001 |
|  | *Ase-ua6* | <0.001 | 0.020 | <0.001 |  |  |  | 0.026 | 0.123 | <0.001 |
|  | *Ase-ua7* | 0.002 | 0.028 | <0.001 |  |  |  | 0.011 | 0.091 | <0.001 |
|  | *Ase-ua8* | <0.001 | 0.020 | <0.001 |  |  |  | <0.001 | 0.054 | <0.001 |
|  | *Ase-ua9* | 0.001 | 0.026 | <0.001 |  |  |  | <0.001 | 0.055 | <0.001 |
|  | *Ase-ua11* | 0.008 | 0.044 | <0.001 | 0.013 | 0.095 | <0.001 | 0.001 | 0.058 | <0.001 |
|  | Age | 0.002 | 0.029 | <0.001 | 0.009 | 0.083 | <0.001 | 0.030 | 0.132 | <0.001 |
|  | Season | 0.003 | 0.033 | <0.001 | <0.001 | 0.052 | <0.001 | 0.019 | 0.110 | <0.001 |
|  | Sex | 0.010 | 0.049 | <0.001 | 0.003 | 0.066 | <0.001 | <0.001 | 0.055 | <0.001 |
|  | Days at 4°C | 0.003 | 0.031 | <0.001 | 0.017 | 0.103 | <0.001 | 0.002 | 0.062 | <0.001 |
|  | Time of day | <0.001 | 0.021 | <0.001 | 0.004 | 0.068 | <0.001 | <0.001 | 0.055 | <0.001 |
|  | Sample Year | 0.013 | 0.076 | 0.006 | 0.043 | 0.206 | 0.024 | 0.021 | 0.178 | 0.018 |

Table S4. The relationship between gut microbiome alpha diversity (richness and Shannon diversity) and variation in host Major histocompatibility complex (MHC) presence/absence of specific (significant in 16S analysis) MHC alleles in adult Seychelles warblers. Generalised linear mixed models with a negative binomial distribution were used for metagenomics taxonomy diversity (N = 99 samples, 57 individuals), and linear mixed models were used for metagenomics functional diversity (N = 99 samples, 57 individuals). Reference categories for categorical variables were as follows: Female (sex), winter (season), 2017 (Sample year), and absent (in all MHC alleles). Significant (P < 0.05) variables are shown in bold.

| Predictors | Metagenomics functional richness | | | | Metagenomics functional Shannon diversity | | | |
| --- | --- | --- | --- | --- | --- | --- | --- | --- |
|  | Est | SE | t | P | Est | SE | t | P |
| (Intercept) | **1.24** | **0.17** | **7.41** | **< 0.001** | **1024.450** | **184.100** | **5.565** | **< 0.001** |
| Heterozygosity |  |  |  |  |  |  |  |  |
| Ase-dab3 |  |  |  |  |  |  |  |  |
| Ase-dab4 |  |  |  |  |  |  |  |  |
| Ase-dab5 |  |  |  |  |  |  |  |  |
| Ase-ua1 |  |  |  |  |  |  |  |  |
| Ase-ua3 |  |  |  |  |  |  |  |  |
| Ase-ua4 |  |  |  |  |  |  |  |  |
| Ase-ua5 |  |  |  |  |  |  |  |  |
| Ase-ua6 |  |  |  |  |  |  |  |  |
| Ase-ua7 |  |  |  |  |  |  |  |  |
| Ase-ua8 |  |  |  |  |  |  |  |  |
| Ase-ua9 |  |  |  |  |  |  |  |  |
| Ase-ua11 | 0.05 | 0.11 | 0.44 | 0.66 | -60.530 | 103.310 | -0.586 | 0.561 |
| Age | **-0.05** | **0.02** | **-2.38** | **0.02** | -41.700 | 21.230 | -1.964 | 0.054 |
| Season (summer) | -0.01 | 0.13 | -0.10 | 0.92 | 125.180 | 141.490 | 0.885 | 0.379 |
| Sex (male) | -0.08 | 0.11 | -0.73 | 0.47 | -8.360 | 107.100 | -0.078 | 0.938 |
| Days at 4°C | -0.07 | 0.10 | -0.68 | 0.50 | 12.680 | 121.900 | 0.104 | 0.917 |
| Time of day | -0.05 | 0.10 | -0.52 | 0.61 | 88.540 | 115.770 | 0.765 | 0.447 |
| Sample Year (2018) | 0.13 | 0.16 | 0.85 | 0.40 | 79.380 | 171.340 | 0.463 | 0.644 |
| Sample Year (2019) | -0.05 | 0.21 | -0.22 | 0.83 | 94.310 | 232.050 | 0.406 | 0.686 |
| Sample Year (2020) | 0.08 | 0.26 | 0.31 | 0.76 | -99.300 | 288.020 | -0.345 | 0.731 |
| Sample Year (2021) | 0.10 | 0.20 | 0.50 | 0.62 | 99.280 | 232.440 | 0.427 | 0.671 |
| Sample Year (2022) | 0.34 | 0.18 | 1.86 | 0.07 | 283.560 | 198.730 | 1.427 | 0.158 |
| Sample Year (2023) | -0.05 | 0.21 | -0.23 | 0.82 | 141.400 | 234.950 | 0.602 | 0.549 |

Table S5. Heterogeneity of variance of categorical variables that were significantly associated with GM composition of the adult Seychelles warblers (Table 2) in of (I) 16S amplicon sequencing variants (ASV) composition, (II) metagenomic taxonomic composition, (III) metagenomic functional gene composition categories.

| (I) 16S Heterogeneity of variance | | | | | | |
| --- | --- | --- | --- | --- | --- | --- |
|  | Df | SumSq | MeanSq | F | N.Perm | P |
| Sample Year | 5 | 288.8 | 57.764 | 1.16 | 999 | 0.327 |
| Season | 1 | 70.7 | 70.655 | 1.381 | 999 | 0.256 |
| Ase-ua5 | 1 | 12 | 12.039 | 0.2246 | 999 | 0.623 |
| Ase-ua7 | 1 | 44.3 | 44.345 | 0.8283 | 999 | 0.363 |
| Ase-ua9 | 1 | 6.7 | 6.691 | 0.1242 | 999 | 0.698 |
| (II) Metagenomic taxonomy Heterogeneity of variance | | | | | | |
| Sample Year | 6 | 192.68 | 32.113 | 0.9845 | 999 | 0.416 |
| Season | 1 | 4.2 | 4.234 | 0.1186 | 999 | 0.743 |
| Ase-ua7 | 1 | 51.8 | 51.772 | 1.4526 | 999 | 0.218 |
| (III) Metagenomic functional Heterogeneity of variance | | | | | | |
| Sample Year | 6 | 8149 | 1358.2 | 0.8667 | 999 | 0.537 |
| Season | 1 | 1304 | 1304.2 | 0.8416 | 999 | 0.37 |

Table S6. PERMANOVA analyses of gut microbiome composition in relation to the presence/absence of all individual major histocompatibility complex (MHC) alleles in adult Seychelles warblers. Performed using Euclidean distance matrices of CLR-transformed abundances of metagenomic functional gene composition categories. Significant predictors (p<0.05) are in bold. N=99 samples from 57 individuals were used for analyses of metagenomic functional composition. Bird ID was included as a blocking factor.

| Predictors | Metagenomics functional gene composition | | | |
| --- | --- | --- | --- | --- |
|  | *df* | R^2^ | F | *p* |
| Heterozygosity |  |  |  |  |
| *Ase-dab3* |  |  |  |  |
| *Ase-dab4* |  |  |  |  |
| *Ase-dab5* |  |  |  |  |
| *Ase-ua1* |  |  |  |  |
| *Ase-ua3* |  |  |  |  |
| *Ase-ua4* |  |  |  |  |
| *Ase-ua5* | 1 | 0.008 | 0.799 | 0.604 |
| *Ase-ua6* |  |  |  |  |
| *Ase-ua7* | 1 | 0.006 | 0.611 | 0.502 |
| *Ase-ua8* |  |  |  |  |
| *Ase-ua9* | 1 | 0.007 | 0.644 | 0.824 |
| *Ase-ua11* |  |  |  |  |
| Age | 1 | 0.011 | 1.080 | 0.796 |
| Season | 1 | 0.015 | 1.421 | 0.278 |
| Sample Year | 6 | 0.062 | 1.011 | 0.354 |
| Sex | 1 | 0.009 | 0.900 | 0.198 |
| Days at 4°C | **1** | **0.013** | **1.297** | **0.014** |
| Time of day | 1 | 0.014 | 1.345 | 0.224 |

Table S7. Differentially abundant eggNOG members with increasing MHC-I Diversity in the gut microbiome of the Seychelles warblers (n = 99 from 57 birds). Categories are COG functional categories: E - Amino acid transport and metabolism, G - Carbohydrate transport and metabolism, I - Lipid transport and metabolism, J – Translation, ribosomal structure and biogenesis, K - Transcription, L - Replication, recombination and repair, M - Cell wall/membrane/envelope biogenesis, O - Posttranslational modification, protein turnover, chaperones, P - Inorganic ion transport and metabolism, R - General function prediction only, T - Signal transduction mechanisms, V - Defense mechanisms.

| **eggNOG members** | **Direction of log fold change** | **Annotation** | **Category** | **KEGG orthologs** | **KEGG ortholog names** | **KEGG pathway mapID** | **KEGG pathways** |
| --- | --- | --- | --- | --- | --- | --- | --- |
| COG0008 | Negative | Glutamyl- or glutaminyl-tRNA synthetase | J | K01885, K01886 | glutamyl-tRNA synthetase [EC:6.1.1.17] & glutaminyl-tRNA synthetase [EC:6.1.1.18] | map00970, map00860, map01100, map01110, map01120, map01240 | Aminoacyl-tRNA biosynthesis; Porphyrin metabolism; Metabolic pathways; Biosynthesis of secondary metabolites; Microbial metabolism in diverse environments; Biosynthesis of cofactors |
| COG0028 | Negative | Acetolactate synthase large subunit or other thiamine pyrophosphate-requiring enzyme | E H | K01652 | acetolactate synthase I/II/III large subunit [EC:2.2.1.6] | map00290, map00650, map00660, map00770, map01210, map01230, map01100, map01110 | Valine, leucine and isoleucine biosynthesis; Butanoate metabolism; C5-Branched dibasic acid metabolism; Pantothenate & CoA biosynthesis; 2-Oxocarboxylic acid metabolism; Biosynthesis of amino acids; Metabolic pathways; Biosynthesis of secondary metabolites |
| COG0085 | Negative | DNA-directed RNA polymerase, beta subunit/140 kD subunit | K | K03043 | DNA-directed RNA polymerase subunit beta [EC:2.7.7.6] | map03020 | RNA polymerase |
| COG0258 | Negative | 5'-3' exonuclease Xni/ExoIX (flap endonuclease) | L | K02335, K04799 | DNA polymerase I [EC:2.7.7.7] & RAD2; flap endonuclease-1 [EC:3.1.-.-] | map03030, map03410, map03420, map03440, map03450 | DNA replication; Base excision repair; Nucleotide excision repair; Homologous recombination; Non-homologous end-joining |
| COG0318 | Negative | O-succinylbenzoic acid-CoA ligase MenE or related acyl-CoA synthetase (AMP-forming) | I | K00666 | fatty-acyl-CoA synthase [EC:6.2.1.-] | - | - |
| COG0443 | Negative | Molecular chaperone DnaK (HSP70) | O | K04043, K03283 | molecular chaperone DnaK & heat shock 70kDa protein 1/6/8 | - | - |
| COG0477 | Negative | MFS family permease, includes anhydromuropeptide permease AmpG | G E P R | None | - | - | - |
| COG0488 | Negative | ATPase components of ABC transporters with duplicated ATPase domains | R | K06158, K15738 | ATP-binding cassette, subfamily F, member 3 & ABC transport system ATP-binding/permease protein | map02010 | ABC transporters |
| COG0507 | Negative | ATPase/5'-3' helicase helicase subunit RecD of the DNA repair enzyme RecBCD (exonuclease V) | L | K03581 | exodeoxyribonuclease V alpha subunit [EC:3.1.11.5] | map03440 | Homologous recombination / DNA repair |
| COG0513 | Negative | Superfamily II DNA and RNA helicase | L | None | - | - | - |
| COG0515 | Negative | Serine/threonine protein kinase | T | K08832, K15409 | serine/threonine-protein kinase SRPK3 [EC:2.7.11.1] & SRPK1 [EC:2.7.11.1] | map05168 | Herpes simplex virus 1 infection |
| COG0524 | Negative | Sugar or nucleoside kinase, ribokinase family | G | K00852, K00847, K00874 | ribokinase [EC:2.7.1.15] & fructokinase [EC:2.7.1.4] & 2-dehydro-3-deoxygluconokinase [EC:2.7.1.45] | map00030, map00051 | Pentose phosphate pathway; Fructose and mannose metabolism |
| COG0577 | Positive | ABC-type antimicrobial peptide transport system, permease component | V | K02004 | putative ABC transport system permease protein | - | - |
| COG0628 | Positive | Predicted PurR-regulated permease PerM | R | None | - | - | - |
| COG0642 | Positive | Signal transduction histidine kinase | T | None | - | - | - |
| COG0697 | Negative | Permease of the drug/metabolite transporter (DMT) superfamily | G E R | None | - | - | - |
| COG0745 | Positive | DNA-binding response regulator, OmpR family, contains REC and winged-helix (wHTH) domain | K T | None | - | - | - |
| COG0841 | Positive | Multidrug efflux pump subunit AcrB | V | K18138 | multidrug efflux pump | map01501, map01503 | Beta-lactam resistance, CAMP (cationic antimicrobial peptide) resistance |
| COG0845 | Positive | Multidrug efflux pump subunit AcrA (membrane-fusion protein) | V M | None | - | - | - |
| COG1502 | Negative | Phosphatidylserine/phosphatidylglycerophosphate/cardiolipin synthase | I | K06131, K01115 | cardiolipin synthase A/B [EC:2.7.8.-] & phospholipase D1/2 [EC:3.1.4.4] | map00564, map00565, map01100, map01110, map04014, map04024, map04072, map04071, map04144, map04666, map04724, map04912, map05200, map05208, map05212, map05231 | Glycerophospholipid metabolism; Ether lipid metabolism; Ras signaling; cAMP signaling; Phospholipase D signaling; Sphingolipid signaling; Endocytosis; Fc gamma R-mediated phagocytosis; Glutamatergic synapse; GnRH signaling; Pathways in cancer |
| COG2911 | Positive | Phospholipid transport to the outer membrane protein TamB | M | K09800 | translocation and assembly module TamB | - | - |
| COG3209 | Positive | Uncharacterized conserved protein RhaS, contains 28 RHS repeats | R | None | - | - | - |
| COG4886 | Negative | Leucine-rich repeat (LRR) protein | K | None | - | - | - |
| COG4974 | Positive | Site-specific recombinase XerD | L | K04763 | integrase/recombinase XerD | - | - |

Table S8. The relationship between gut microbiome relative abundance (CLR transformed) of A) microbial defence and B) metabolism gut microbiome significant functional categories and variation in host MHC-I diversity in Seychelles warblers. Linear mixed models were used for all models. N = 99 samples from 57 individuals. Reference categories for categorical variables were as follows: winter (season), 2017 (Sample year). Significant (P < 0.05) variables are shown in bold.

|  | A) Microbial defence genes abundance | | | | | B) Microbial metabolism genes abundance | | | | |
| --- | --- | --- | --- | --- | --- | --- | --- | --- | --- | --- |
|  | Estimate | SE | df | t | P | Estimate | SE | df | t | P |
| (Intercept) | -2.27 | 1.35 | 52.24 | -1.68 | 0.099 | 3.52 | 0.81 | 54.49 | 4.35 | 0.000 |
| **MHC-I Diversity** | **0.32** | **0.13** | **42.88** | **2.48** | **0.017** | **-0.26** | **0.08** | **45.65** | **-3.38** | **0.001** |
| MHC-II Diversity | 0.12 | 0.17 | 44.12 | 0.70 | 0.486 | -0.11 | 0.10 | 46.60 | -1.09 | 0.281 |
| Heterozygosity | 0.48 | 0.90 | 50.51 | 0.53 | 0.599 | 0.57 | 0.54 | 52.25 | 1.05 | 0.300 |
| Season (summer) | 0.43 | 0.44 | 83.19 | 0.97 | 0.333 | -0.08 | 0.26 | 85.42 | -0.32 | 0.753 |
| Sample Year (2018) | -0.24 | 0.50 | 85.37 | -0.49 | 0.626 | -0.24 | 0.29 | 84.10 | -0.84 | 0.402 |
| Sample Year (2019) | -0.20 | 0.69 | 85.15 | -0.29 | 0.776 | -0.30 | 0.40 | 83.34 | -0.75 | 0.458 |
| Sample Year (2020) | -0.16 | 0.86 | 82.67 | -0.19 | 0.853 | -0.21 | 0.49 | 79.30 | -0.42 | 0.675 |
| Sample Year (2021) | 0.25 | 0.63 | 59.28 | 0.40 | 0.688 | 0.05 | 0.35 | 56.52 | 0.15 | 0.884 |
| Sample Year (2022) | 0.04 | 0.57 | 82.72 | 0.07 | 0.941 | -0.25 | 0.33 | 80.74 | -0.77 | 0.444 |
| Sample Year (2023) | -1.61 | 0.64 | 74.13 | -2.53 | 0.014 | -0.23 | 0.36 | 71.71 | -0.64 | 0.522 |
| **Days at 4°C** | **1.14** | **0.34** | **72.95** | **3.33** | **0.001** | **-0.45** | **0.19** | **70.88** | **-2.30** | **0.025** |

Table S9. Permutation tests of differences between low and high MHC diversity on metagenomic gut microbiome networks in adult Seychelles warblers. Performed using (I) metagenomic taxonomic network, (II) metagenomic functional gene network with separate models for low and high (A) MHC-I diversity and (B) MHC-II diversity. Significant predictors (p<0.05) are in bold.

|  | (I) Metagenomic taxonomic networks | | (I) Metagenomic functional networks | |
| --- | --- | --- | --- | --- |
| (A) MHC-I |  |  |  |  |
|  | Absolute difference | P | Absolute difference | P |
| Edge density | 0.006 | 0.485 | **0.016** | **0.040** |
| Modularity | 0.056 | 0.812 | 0.107 | 0.178 |
| Positive edge percentage | 0.980 | 0.772 | 13.639 | 0.168 |
| (B) MHC-II |  |  |  |  |
| Edge density | 0.001 | 0.921 | 0.006 | 0.574 |
| Modularity | 0.063 | 0.851 | 0.003 | 0.990 |
| Positive edge percentage | 10.909 | 0.515 | 9.168 | 0.663 |

## MHC divergence versus MHC diversity

Methods

We tested whether MHC divergence explained more variation in GM characteristics than MHC diversity, as suggested by the reviewers. We constructed complementary alpha diversity models (16S metabarcoding, metagenomic taxonomic, and metagenomic functional) in which MHC divergence replaced MHC diversity. Model performances were compared using Akaike Information Criterion (AIC) values calculated with the *AIC*() function. An AIC difference >7 between two models is considered evidence for improved model fit (Burnham et al., 2011).

To assess whether MHC divergence explained more variation in GM composition than MHC diversity, we constructed alternative PERMANOVA models (16S metabarcoding, metagenomic taxonomic, and metagenomic functional) in which MHC divergence replaced MHC diversity. Model performances were compared using the R² values from the PERMANOVA outputs and Akaike Information Criterion (AIC) values calculated with the *AICc_permanova2*() function from the *AICcPermanova* 0.02 (Corcoran, 2023).

Results

### *16S rRNA metabarcoding diversity*

Model comparisons show that using MHC divergence instead of MHC diversity did not significantly improve model fit (AIC = 3107.7 vs. 3104.6, respectively) for GM richness. However, MHC divergence (rather than MHC diversity) produced a better model fit (AIC = 849.8 vs. 863.1, respectively) for GM Shannon diversity. However, within both models, neither MHC-I nor MHC-II divergence were significantly associated with any measure of 16S GM alpha diversity (Table S10), as was the case for MHC diversity measures.

### *Metagenomic taxonomic diversity*

Model comparisons show that using MHC divergence instead of MHC diversity did not significantly improve model fit (AIC = 827.1 vs. 830.2, respectively) for metagenomic taxonomic richness. However, in the model, taxonomic richness was significantly negatively associated with MHC-I divergence but not MHC-II divergence (Table S10). This finding differs from the model using MHC-I diversity, where it was not significant (Table 2). Using MHC divergence (rather than diversity) did produce a better model fit (AIC = 258.9 vs. 275.6, respectively) for taxonomic Shannon diversity. However, metagenomic taxonomic Shannon diversity was not associated with either MHC-I or MHC-II divergence (Table S10). Thus, the evidence as to whether MHC-I divergence is more important in shaping metagenomic taxonomy than MHC-I diversity remains equivocal.

### *Metagenomic functional diversity*

Model comparisons show that using MHC divergence instead of MHC diversity significantly improved model fit (AIC = 156.6 vs. 168.1, respectively) for functional richness and (AIC = 1264.9 vs. 1279.5, respectively) for functional Shannon diversity. However, within the models, functional alpha diversity was not significantly associated with either MHC-I or MHC-II divergence (Table S10), as was the case for MHC diversity measures.

Table S10. The relationship between gut microbiome alpha diversity (richness and Shannon diversity) and variation in host MHC divergence in adult Seychelles warblers. Generalised linear mixed models with a negative binomial distribution were used for 16S ASV diversity (N = 253 samples from 149 individuals) and metagenomics taxonomy diversity (N = 99 samples, 57 individuals), and linear mixed models were used for metagenomics functional diversity (N = 99 samples, 57 individuals). Reference categories for categorical variables were as follows: Female (sex), winter (season), 2017 (Sample year), and absent (in all MHC alleles). Significant (P < 0.05) variables are shown in bold.

| Predictors | 16S ASV diversity | | | | Metagenomics taxonomic diversity | | | | Metagenomics functional diversity | | | |
| --- | --- | --- | --- | --- | --- | --- | --- | --- | --- | --- | --- | --- |
|  | Est | SE | z | P | Est | SE | z | P | Est | SE | t | P |
| A) Richness |  |  |  |  |  |  |  |  |  |  |  |  |
| (Intercept) | **5.76** | **0.50** | **11.62** | **< 0.001** | **4.64** | **0.93** | **5.01** | **< 0.001** | **1.60** | **0.65** | **2.48** | **0.017** |
| Heterozygosity | -0.15 | 0.21 | -0.73 | 0.465 | 0.47 | 0.43 | 1.10 | 0.273 | -0.11 | 0.29 | -0.39 | 0.699 |
| MHC-I Divergence | -1.64 | 2.29 | -0.72 | 0.474 | **-8.70** | **4.09** | **-2.13** | **0.034** | -0.74 | 2.91 | -0.26 | 0.800 |
| MHC-II Divergence | 0.01 | 0.63 | 0.01 | 0.991 | -1.81 | 1.24 | -1.45 | 0.146 | -0.26 | 0.87 | -0.30 | 0.768 |
| Age | -0.03 | 0.02 | -1.47 | 0.142 | -0.04 | 0.03 | -1.40 | 0.162 | **-0.05** | **0.02** | **-2.30** | **0.025** |
| Season (summer) | 0.04 | 0.12 | 0.34 | 0.732 | 0.22 | 0.21 | 1.01 | 0.313 | -0.01 | 0.14 | -0.08 | 0.933 |
| Sex (male) | **-0.23** | **0.09** | **-2.68** | **0.007** | 0.17 | 0.17 | 1.04 | 0.297 | -0.09 | 0.12 | -0.75 | 0.456 |
| Days at 4°C | -0.01 | 0.10 | -0.13 | 0.894 | 0.02 | 0.18 | 0.10 | 0.923 | -0.08 | 0.10 | -0.73 | 0.469 |
| Time of day | 0.02 | 0.09 | 0.27 | 0.791 | **0.36** | **0.17** | **2.04** | **0.041** | -0.05 | 0.10 | -0.48 | 0.630 |
| Sample Year (2018) | -0.01 | 0.10 | -0.07 | 0.941 | 0.05 | 0.27 | 0.20 | 0.842 | 0.12 | 0.16 | 0.77 | 0.443 |
| Sample Year (2019) | -0.04 | 0.14 | -0.27 | 0.788 | -0.29 | 0.37 | -0.80 | 0.425 | -0.06 | 0.22 | -0.26 | 0.798 |
| Sample Year (2020) | 0.12 | 0.17 | 0.68 | 0.497 | -0.24 | 0.45 | -0.54 | 0.586 | 0.06 | 0.26 | 0.23 | 0.822 |
| Sample Year (2021) | **0.45** | **0.22** | **2.07** | **0.039** | 0.08 | 0.36 | 0.22 | 0.824 | 0.10 | 0.20 | 0.49 | 0.624 |
| Sample Year (2022) | 0.23 | 0.16 | 1.41 | 0.160 | 0.47 | 0.31 | 1.54 | 0.123 | 0.32 | 0.19 | 1.74 | 0.086 |
| Sample Year (2023) |  |  |  |  | 0.17 | 0.36 | 0.47 | 0.640 | -0.06 | 0.22 | -0.29 | 0.776 |
| B) Shannon diversity |  |  |  |  |  |  |  |  |  |  |  |  |
| (Intercept) | **3.55** | **0.95** | **3.75** | **< 0.001** | **2.70** | **1.16** | **2.34** | **0.024** | **1360.00** | **628.75** | **2.16** | **0.035** |
| Heterozygosity | 0.07 | 0.40 | 0.17 | 0.863 | 0.61 | 0.53 | 1.16 | 0.252 | 94.96 | 286.03 | 0.33 | 0.741 |
| MHC-I Divergence | -0.28 | 4.37 | -0.07 | 0.948 | -9.39 | 5.15 | -1.83 | 0.075 | -2172.24 | 2809.09 | -0.77 | 0.443 |
| MHC-II Divergence | -0.43 | 1.21 | -0.36 | 0.719 | -1.71 | 1.56 | -1.09 | 0.282 | -432.60 | 837.39 | -0.52 | 0.608 |
| Age | -0.03 | 0.03 | -0.81 | 0.421 | -0.02 | 0.04 | -0.65 | 0.519 | -41.87 | 21.51 | -1.95 | 0.056 |
| Season (summer) | -0.15 | 0.22 | -0.68 | 0.497 | 0.02 | 0.24 | 0.07 | 0.944 | 157.42 | 145.55 | 1.08 | 0.283 |
| Sex (male) | -0.30 | 0.17 | -1.80 | 0.075 | 0.15 | 0.21 | 0.70 | 0.486 | -8.61 | 111.21 | -0.08 | 0.939 |
| Days at 4°C | -0.10 | 0.18 | -0.57 | 0.567 | -0.27 | 0.19 | -1.43 | 0.156 | 23.53 | 123.18 | 0.19 | 0.849 |
| Time of day | -0.02 | 0.16 | -0.14 | 0.890 | 0.14 | 0.19 | 0.78 | 0.440 | 104.70 | 118.39 | 0.88 | 0.379 |
| Sample Year (2018) | 0.16 | 0.24 | 0.67 | 0.502 | 0.19 | 0.30 | 0.64 | 0.525 | 88.47 | 172.76 | 0.51 | 0.610 |
| Sample Year (2019) | -0.11 | 0.30 | -0.37 | 0.712 | -0.20 | 0.40 | -0.50 | 0.622 | 89.03 | 240.73 | 0.37 | 0.713 |
| Sample Year (2020) | 0.46 | 0.39 | 1.18 | 0.239 | 0.29 | 0.48 | 0.61 | 0.546 | -97.64 | 294.33 | -0.33 | 0.741 |
| Sample Year (2021) | -0.16 | 0.30 | -0.52 | 0.603 | 0.14 | 0.37 | 0.37 | 0.715 | 108.32 | 238.02 | 0.46 | 0.650 |
| Sample Year (2022) | 0.13 | 0.29 | 0.44 | 0.662 | 0.30 | 0.34 | 0.88 | 0.381 | 282.64 | 201.02 | 1.41 | 0.164 |
| Sample Year (2023) |  |  |  |  | 0.46 | 0.39 | 1.17 | 0.245 | 126.95 | 243.94 | 0.52 | 0.604 |

### *16S rRNA metabarcoding composition*

Model comparison showed that using MHC divergence rather than MHC diversity did not significantly improve model fit (AIC = 1810.8 vs. 1811.5, respectively), and MHC divergence did not explain more variance (Table 2A, Table S11). Within the models, 16S GM composition was significantly associated with MHC-II (but not MHC-I) divergence (Table S11). However, 16S GM composition was significantly associated with both MHC-I and MHC-II diversity (Table 2A).

### *Metagenomic taxonomic composition*

Model comparison showed that using MHC divergence rather than MHC diversity did not significantly improve model fit (AIC = 621.8 vs. 621.2, respectively), and MHC divergence did not explain more variance (Table 2A, Table S11). Metagenomic taxonomic composition was not associated with either class I or class II MHC divergence (Table S11).

### *Metagenomics functional composition*

Model comparison showed that using MHC divergence rather than MHC diversity did not significantly improve model fit (AIC = 967.4 vs. 966.7, respectively), and MHC divergence did not explain more variance (Table 2A, Table S11). Metagenomic functional composition was not associated with either class I or class II MHC divergence (Table S11).

Table S11. PERMANOVA analyses of gut microbiome composition in relation to individual MHC divergence characteristics in adult Seychelles warblers. Performed using Euclidean distance matrices of CLR-transformed abundances of (I) 16S ASV composition, (II) metagenomic taxonomic composition, (III) metagenomic functional gene composition categories. Significant predictors (p<0.05) are in bold. N = 253 samples from 149 individuals were included in the 16S metabarcoding analyses, N = 99 samples from 57 individuals were used for analyses of metagenomic taxonomic and functional composition. Bird ID was included as a blocking factor.

| Predictor | (I) 16S ASV composition | | | | (I) Metagenomics taxonomic composition | | | | | | (II) Metagenomics functional gene composition | | | | | |
| --- | --- | --- | --- | --- | --- | --- | --- | --- | --- | --- | --- | --- | --- | --- | --- | --- |
|  | *df* | R^2^ | F | *p* | *df* | R^2^ | F | | *p* | | *df* | R^2^ | F | | *p* | |
| Heterozygosity | 1 | 0.003 | 0.722 | 0.429 | 1 | 0.007 | | 0.783 | | 0.315 | 1.000 | 0.013 | | 1.342 | | 0.159 |
| MHC-I Divergence | 1 | 0.006 | 1.631 | 0.499 | 1 | 0.007 | | 0.786 | | 0.305 | 1.000 | 0.013 | | 1.282 | | 0.375 |
| MHC-II Divergence | **1** | **0.004** | **1.141** | **0.017** | 1 | 0.007 | | 0.694 | | 0.793 | 1.000 | 0.004 | | 0.429 | | 0.792 |
| Age | 1 | 0.004 | 0.940 | 0.864 | 1 | 0.017 | | 1.761 | | 0.261 | 1.000 | 0.012 | | 1.172 | | 0.767 |
| Season | 1 | 0.007 | 1.963 | 0.000 | **1** | **0.018** | | **1.908** | | **0.001** | 1.000 | 0.014 | | 1.402 | | 0.093 |
| Sample Year | 5 | 0.039 | 2.079 | 0.000 | **6** | **0.081** | | **1.417** | | **<0.001** | 6.000 | 0.066 | | 1.091 | | 0.221 |
| Sex | 1 | 0.003 | 0.921 | 0.822 | **1** | **0.013** | | **1.414** | | **0.006** | 1.000 | 0.012 | | 1.167 | | 0.360 |
| Days at 4°C | 1 | 0.009 | 2.408 | 0.013 | 1 | 0.010 | | 1.002 | | 0.665 | **1.000** | **0.013** | | **1.313** | | **0.010** |
| Time of day | 1 | 0.010 | 2.656 | 0.001 | **1** | **0.018** | | **1.856** | | **0.002** | 1.000 | 0.016 | | 1.635 | | 0.138 |

Table S12. Table 3. Comparison between MHC variables in models testing how using MHC divergence instead of MHC diversity affected the association between MHC characteristics and the gut microbiome of adult Seychelles warblers. Model tables for GM alpha diversity are in Table S10 (MHC divergence), and Table 1 (MHC diversity), and GM composition is in Table S11 (MHC divergence) and Table 2 (MHC diversity).

| Model | MHC divergence vs. MHC diversity |
| --- | --- |
| GM alpha diversity (Table S10 vs Table 1) | |
| 16S rRNA metabarcoding richness | No difference |
| 16S rRNA metabarcoding Shannon diversity | No difference |
| Metagenomic taxonomic richness | MHC-I divergence negatively associated  MHC-I diversity not associated |
| Metagenomic taxonomic Shannon diversity | No difference |
| Metagenomic functional richness | No difference |
| Metagenomic functional Shannon diversity | No difference |
| GM composition (Table S11 vs Table 2) | |
| 16S rRNA metabarcoding composition | MHC-I divergence not associated  MHC-I diversity associated |
| Metagenomic taxonomic composition | No difference |
| Metagenomic functional composition | No difference |


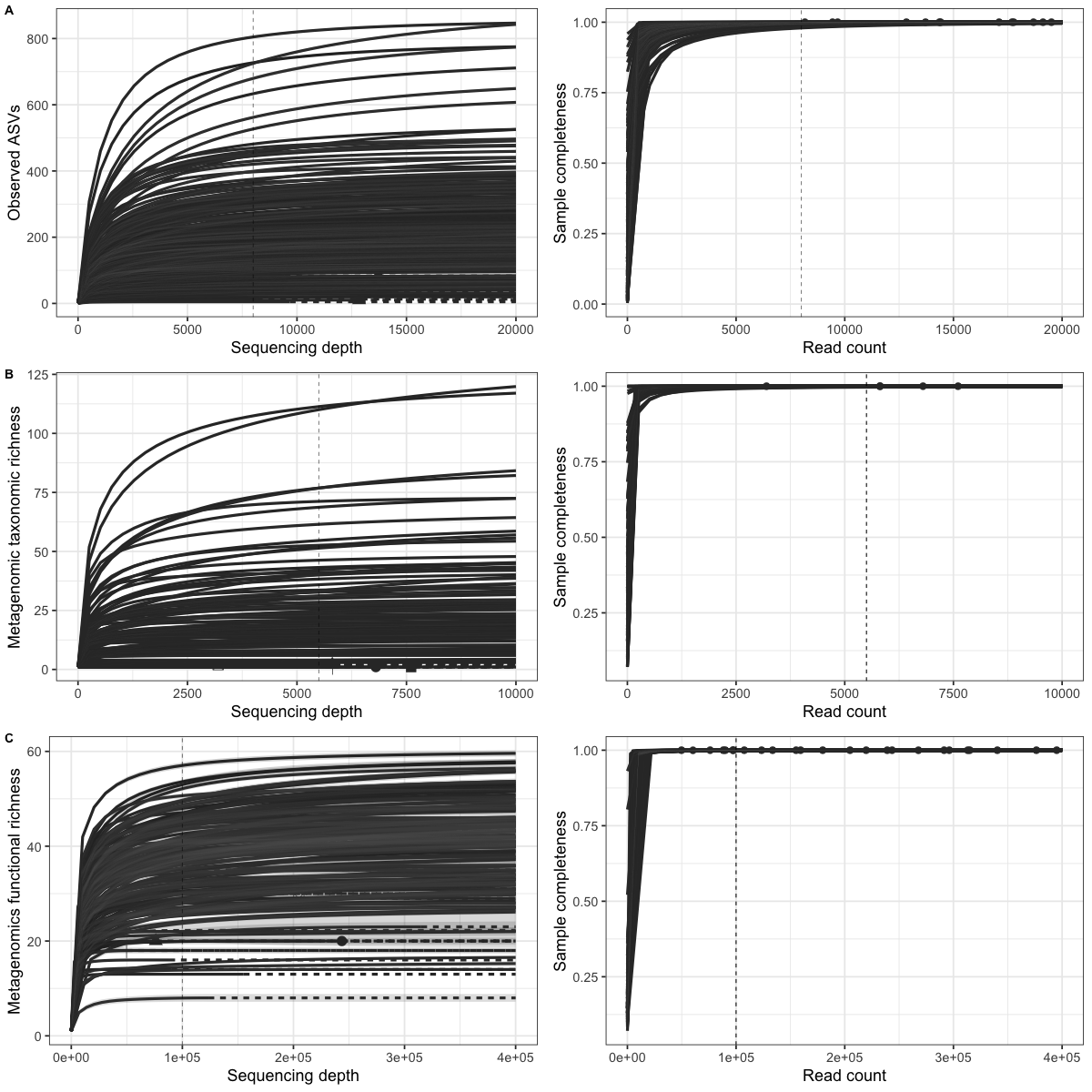


Figure S1. Rarefaction curve of 16S gut microbiome sequencing of the Seychelles warbler.


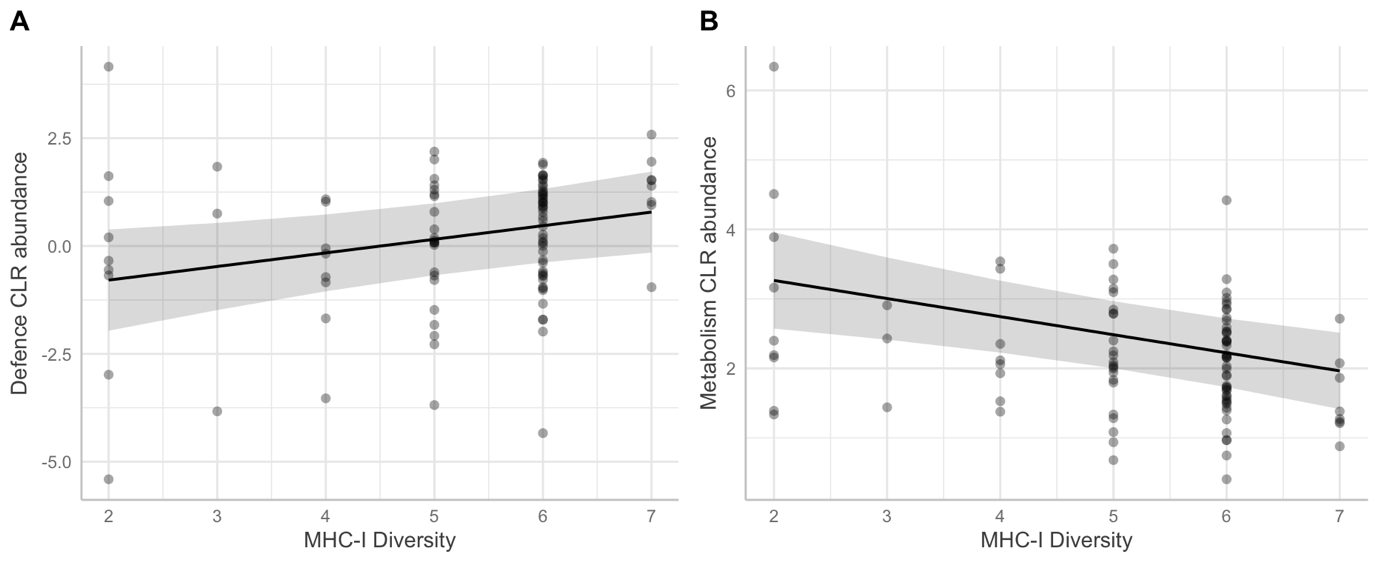


Figure S2. The relationship between gut microbiome relative abundance (CLR transformed) of A) microbial defence and B) metabolism gut microbiome significant functional categories and variation in host MHC-I diversity in Seychelles warblers. Lines and standard error shading are model predictions (Table S8), and points are raw data. N = 99 samples from 57 individuals.
